# Metabolomic Responses of Arabidopsis Suspension Cells to Bicarbonate under Light and Dark Conditions

**DOI:** 10.1038/srep35778

**Published:** 2016-10-20

**Authors:** Biswapriya B. Misra, Zepeng Yin, Sisi Geng, Evaldo de Armas, Sixue Chen

**Affiliations:** 1 Department of Biology, Genetics Institute, Plant Molecular and Cellular Biology Program, University of Florida, Gainesville, FL 32610, USA; 2 Alkali Soil Natural Environmental Science Center, Northeast Forestry University, Key Laboratory of Saline-alkali Vegetation Ecology Restoration in Oil Field, Ministry of Education, Harbin 150040, China; 3 Training Institute, Thermo Fisher Scientific, 1400 North point Parkway, Ste 10., West Palm Beach, FL 33407, USA; 4 Interdisciplinary Center for Biotechnology Research, University of Florida, Gainesville, FL 32610, USA

## Abstract

Global CO_2_ level presently recorded at 400 ppm is expected to reach 550 ppm in 2050, an increment likely to impact plant growth and productivity. Using targeted LC-MS and GC-MS platforms we quantified 229 and 29 metabolites, respectively in a time-course study to reveal short-term responses to different concentrations (1, 3, and 10 mM) of bicarbonate (HCO_3_^−^) under light and dark conditions. Results indicate that HCO_3_^−^ treatment responsive metabolomic changes depend on the HCO_3_^−^ concentration, time of treatment, and light/dark. Interestingly, 3 mM HCO_3_^−^ concentration treatment induced more significantly changed metabolites than either lower or higher concentrations used. Flavonoid biosynthesis and glutathione metabolism were common to both light and dark-mediated responses in addition to showing concentration-dependent changes. Our metabolomics results provide insights into short-term plant cellular responses to elevated HCO_3_^−^ concentrations as a result of ambient increases in CO_2_ under light and dark.

Photosynthesis in green plants involves CO_2_ fixation. The rising atmospheric CO_2_ concentration over the past 150 years has posed significant effects on plant metabolism, physiology and productivity^1^,^2^,^3^. Elevated CO_2_ increases leaf area and number, branching, plant size, biomass, growth rates, C: N ratio and non-structural carbohydrates, in addition to decreased N-compounds such as amino acids and reduced allocation to phenolic compounds in long term^4^. In many C3 plant species, the effects of elevated CO_2_ in the long-term may result in enhanced photosynthesis, but little information are available on short-term plant cellular responses to CO_2_ changes at the metabolomic scale.

Many studies have focused on harnessing the potential of -omics platforms to provide insights into aspects of plant cell metabolism and physiology in response to CO_2_ or light conditions. Omics tools enable systemic view of cellular processes for investigation of metabolic networks and regulatory mechanisms. For instance, transcriptomic studies conducted in *Arabidopsis thaliana*^5^,^6^, rice (*Oryza sativa*)^7^, and *Populus euramericana*^8^ have provided meaningful insights into the molecular basis of plant response to elevated CO_2._ A recent study conducted in *Physcomitrella patens* reported transcriptomic changes under elevated CO_2_ conditions, indicating a genome-scale pathway changes^9^. Although it is still rare, proteomic studies of plant elevated CO_2_ responses were conducted in *Aster tripolium*^10^ and *Rumex obtusifolius*^11^, which indicated high expression of antioxidative enzymes and the involvement of organic acids.

Similarly, several studies have focused on the diurnal regulation of plant cellular metabolism. In photosynthetic organisms, the daily light/dark cycle is a major environmental regulator as they rely on solar energy to drive anabolic processes to store the fixed carbon or its catabolism during the dark period. Other light controlled processes include cell division, stress sensitivity, chemotaxis, nutrient uptake and phototaxis^12^. Light-intensity induced changes in *A. thaliana* transcriptome indicated that 20% of transcripts were H_2_O_2_- and ABA-responsive within 20–60 s of light stress^13^. Currently, metabolomic studies of plant diurnal processes are limited. In seagrass *Zostera marina*, the diurnal metabolomic changes were recorded in anoxic conditions^14^. Following an integrative approach of combining transcriptomic and metabolomic datasets into *in silico* metabolic models in rice, light-specific metabolic changes were observed^15^. Although HCO_3_^−^-responsive guard and mesophyll cell responses were cataloged in canola leaves^16^, nothing is yet known about the temporal metabolomic changes associated with differential HCO_3_^−^ levels or under light and dark conditions.

The use of single-cell types eliminates the ‘averaging effect’ of metabolomes that occurs with tissues, organs or whole plants^17^. In one such study using *Chlamydomonas reinhardtii* cells, 128 metabolites with significant differences between high- and low-CO_2_ treated cells were detected, of which 82 included amino acids, lipids, and carbohydrates^18^. Recently, *C. reinhardtii* transcriptome and proteome were studied under varying CO_2_ concentrations. Furthermore, it was observed that varying CO_2_ concentrations had an effect on 25% of the transcriptome in *C. reinhardtii*^19^. Proteomic studies revealed the role of 22 extracellular proteins, which were only expressed under low CO_2_ conditions^20^. In addition, transcriptional coordination of physiological responses in *Nannochloropsis oceanica* CCMP1779, a marine unicellular alga was recorded under light/dark cycles^12^. In another unicellular dinoflagellate (*Symbiodinium sps*.), the transcriptomic changes of cells in response to light intensity under different trophic conditions were investigated^21^. Furthermore, the diurnal transcriptome of *C. reinhardtii* undergoing cellular and metabolomic differentiation was captured^22^. Here we chose NaHCO_3_ treatment as a CO_2_ enrichment model system to investigate its effect on *Arabidopsis* cellular metabolome under varying concentration, duration of exposure, and light and dark conditions.

## Results

### The dark and light responsive metabolomes and their responses to HCO_3_^−^ treatment

Our targeted high performance liquid chromatography (HPLC)-multiple reaction monitoring mass spectrometry (MRM-MS) approach allowed us to detect and quantify a total of 229 metabolites in *A. thaliana* suspension cells at various time-points across four biological replicates (Supplementary Tables S1–3). The polar to semi-polar metabolites covered various pathways of secondary metabolites, amino acids, purines, flavonoids, 2-oxocarboxylic acids, central carbon metabolites, glutathione, and beta-alanine metabolism that were spread over the KEGG metabolic map (Supplementary Figure S1). Another 29 metabolites were identified and quantified using the gas chromatography (GC) platform to include cyanoamino acid, pantothenate, fatty acids, beta-alanine, galactose, glycolysis, branched-chain amino acid, arginine, and proline metabolism among others (Supplementary Table S4).

**Figure 1:**
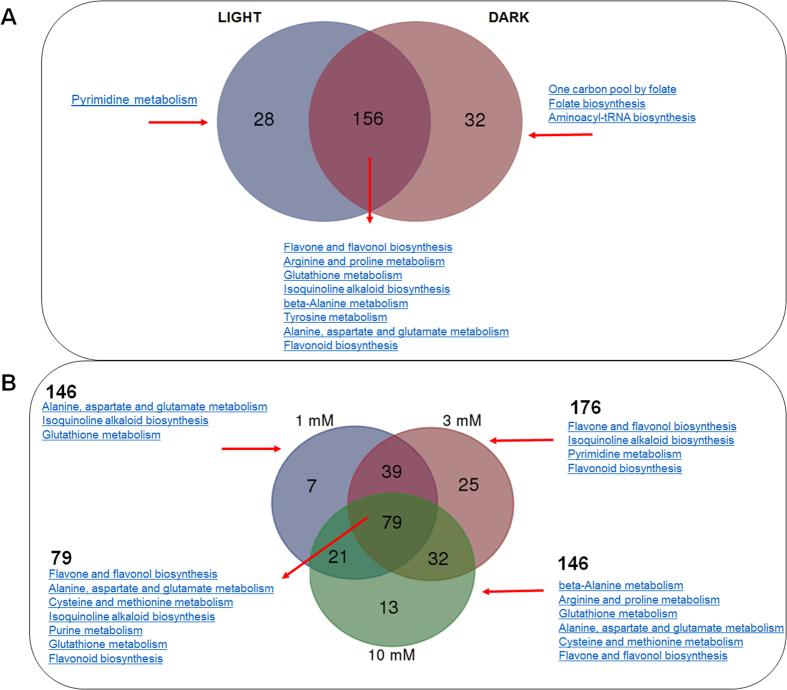
Light and HCO_3_^−^ concentration-dependent common and unique metabolites during the treatment. (**A**) A two-way Venn diagram showing common and unique metabolites in light and dark conditions. (**B**) A three-way Venn diagram showing common and unique metabolites in different concentrations (1, 3, and 10 mM) in the time-course study.

The HCO_3_^−^ treated suspension cell metabolomes showed quantitative variations based on HCO_3_^−^ concentrations (1, 3, and 10 mM), light and dark, and the time of treatment. Across the treatments, the shared metabolites showed differential levels of changes (fold change cut-offs of <0.8 and >1.2 at *p* < 0.05) (Supplementary Table S3). The significantly changing metabolites revealed 156 changing metabolites (either increase or decrease), which are common to both light and dark conditions (Fig. 1A). These common metabolites are enriched in flavones, flavonol, and flavonoid biosynthesis, amino acid (Arg, Pro, Tyr, Ala, Asp and Glu) metabolism, alkaloid biosynthesis and glutathione metabolism. On the other hand, 28 significantly changed metabolites are enriched in pyridine biosynthesis and 32 in one carbon pool by folate, folate biosynthesis and amino acid metabolism. Concentration dependent metabolomic changes revealed shared pathways (i.e., flavonoid, alkaloid, glutathione metabolism), with 3 mM treatment induced the highest number (176) of significant changes enriched in pyrimidine and flavonoid metabolism (Fig. 1B). For 10 mM treatment, beta-alanine, Arg and Pro and Cys and Met metabolism showed enrichment. In addition, the commonly enriched pathways based on 79 commonly shared metabolites showed concentration-independent changes belong to flavone, flavonol and other flavonoid biosynthesis, purine metabolism, glutathione metabolism and amino acid metabolism (Ala, Asp, Glu, Cys, and Met) (Fig. 1B).

### Grouped metabolomic changes in HCO_3_^−^ treated cells

Within subject-ANOVA analysis using the four factors including treatment (control vs. HCO_3_^−^), light conditions (light and dark), concentration (1, 3, and 10 mM), time-course (0–120 min) and interactions indicated significant changes (*p* < 0.05) in 89, 123, 228, 112 and 28 metabolites, respectively (Supplementary Table S5). Taurine metabolism was significantly affected by time, whereas flavonoid biosynthesis, glutathione and phenylalanine metabolism were associated with light dependent changes. Furthermore, both taurine metabolism and flavonoid metabolism were associated with HCO_3_^−^ treatment-specific changes, whereas glyoxylate and dicarboxylate metabolism, Ala, Asp and Glu metabolism and taurine metabolism were responsive to interactions of all factors (Table 1). However, the highest numbers of metabolites were enriched in flavonoid biosynthesis, glutathione metabolism, beta-Ala metabolism, purine and pyrimidine metabolism, Phe and Tyr metabolism.

**Figure 2:**
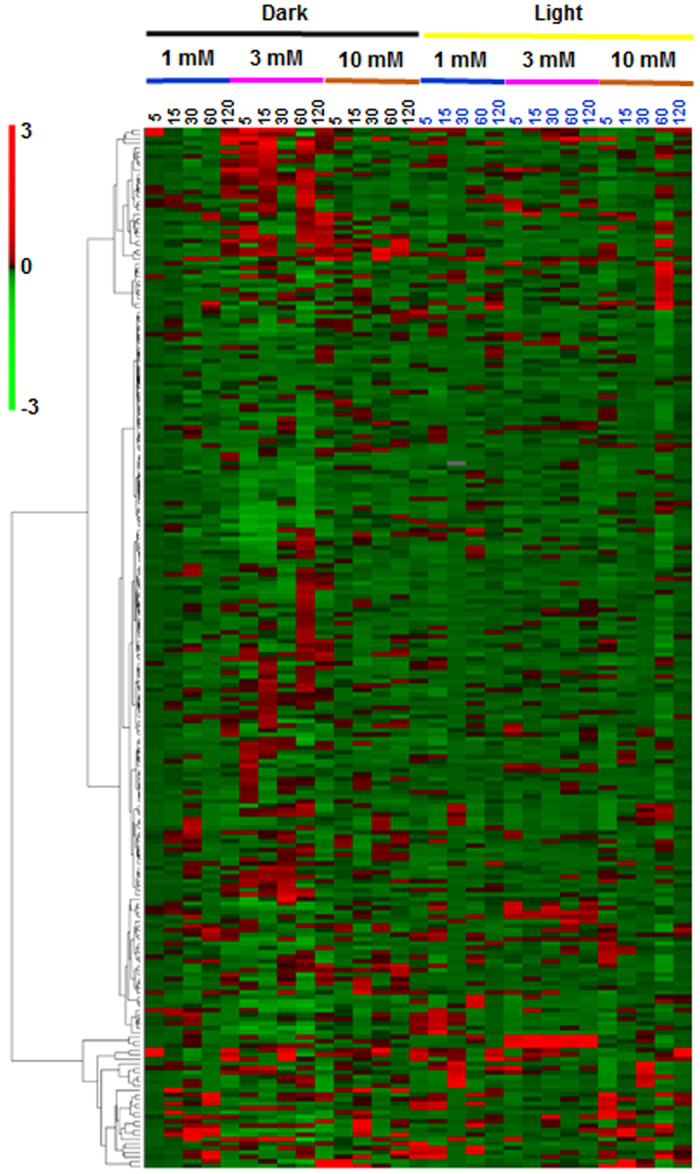
Hierarchical cluster analysis (HCA) analysis of the mean values of metabolite contents. Results are from four biological replicates showing 229 metabolites common to all the treatments depicting the data structure dependent on light, concentrations, and time course (0–120 min) of HCO_3_^−^ treatment. Red and green indicate high and low concentrations of metabolites, respectively. Values were subjected to average linkage clustering (Euclidean distance).

**Figure 3:**
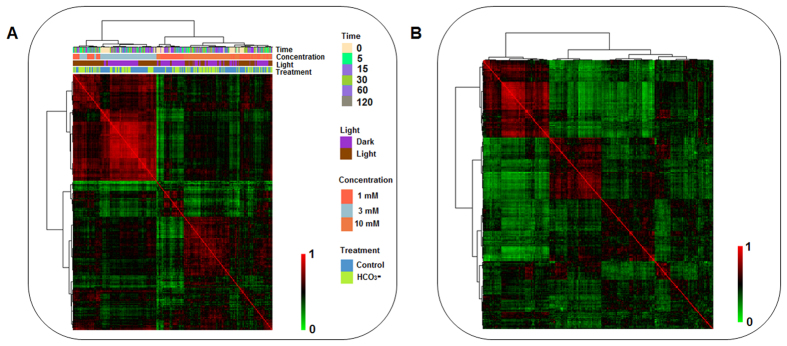
Correlation among samples and metabolites in the study. (**A**) Samples pair-wise correlation heat map for control and HCO_3_^−^ treated time-course profiling study (0, 5, 15, 30 60, 120 min). Columns and rows refer to the samples represented as a function of metabolites. Clustering on correlation coefficients demonstrate the grouping of samples based on their ‘metabotype’. (**B**) Metabolites pair-wise correlation heat map for control and HCO_3_^−^ treated time-course profiling study (0, 5, 15, 30 60, 120 min). Columns and rows refer to metabolites arranged based on the Pearson correlation co-efficient. Highly correlated (red) metabolites in both cell-types belong to sugars and amino acids as compared to the lowly correlated ones (green).

Hierarchical clustering analysis (HCA), performed to classify all metabolites based on treatments, light conditions, concentrations, and time-points revealed three distinct clusters (Fig. 2). The first cluster mostly reflected the increased metabolites under the dark conditions, while the last cluster reflected increased metabolites across all the concentrations and conditions. Similarly, when all samples were clustered by HCA as a function of metabolite responses, they were clustered into several regions of high and low correlations (Spearman) (Fig. 3A). For 3 mM treatment, all the samples under dark and light conditions correlated well among each other (R^2^ < 0.9) in a condition specific manner, i.e., light-independent HCO_3_^−^ response, whereas for 1 mM and 10 mM treatments, the correlations were low (Fig. 3A). Similarly, metabolite pair-wise (Spearman) correlation map indicated strong correlation among sugar and nucleotide phosphates and amino acids during the time-course study (Fig. 3B).

### Temporal metabolomic changes in HCO_3_^−^ treated cells

**Figure 4:**
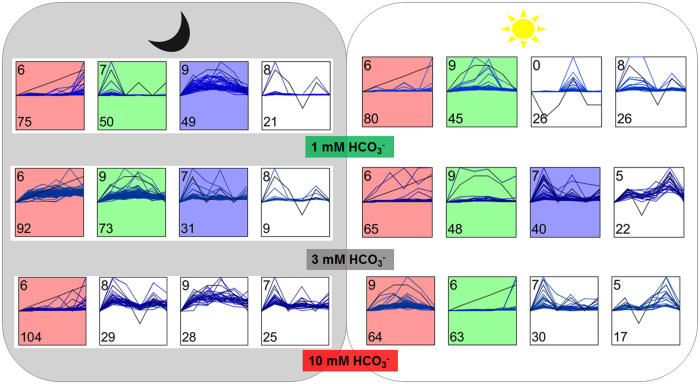
Short Time series Expression Miner (STEM) analysis displaying patterns of metabolite changes in dark and light conditions across the HCO_3_^−^ treatment concentrations. The numbers in the bottom left corner indicate number of metabolites following the pattern, while the numbers on top left indicate the serial number of the predicted model out of the 10 generated models, where only the top four models for each conditions are shown.

Short time-series expression miner (STEM) analyses showed that under both light and dark conditions the most significant pattern (*p* < 0.05) was a consistent increase (upward trend) throughout the time-course (Fig. 4). In fact, under dark conditions the number of metabolites displaying this pattern was directly proportional to the 1, 3, and 10 mM HCO_3_^−^concentrations, with 75, 92 and 104 metabolites, respectively (Supplementary Table S6). Pathways enriched in the dark across the three concentrations include purine and pyrimidine metabolism, flavonoid biosynthesis, beta-alanine metabolism, tyrosine metabolism and alkaloid metabolism. This is completely opposite to what was observed under the light conditions, where 80, 65 and 63 metabolites (enriched with flavone, flavonol and other flavonoid biosynthesis, alkaloid metabolism, and tyrosine metabolism) showed increasing patterns with 1, 3, and 10 mM HCO_3_^−^, respectively. In contrast, the number of metabolites for the other two-patterns (i.e., bell-shaped with initial increase followed by decrease in later time-points and two-peaked patterns) showed consistent decreases. The bell-shaped pattern showed concentration-dependent increases in the number of metabolites i.e., 45, 48, and 64 metabolites for 1, 3, and 10 mM HCO_3_^−^, respectively, usually enriched with beta-alanine metabolism (Supplementary Table S6). While a few of the models demonstrated clear and linear patterns of changes, a majority of the metabolites showed mostly biphasic patterns with two peaks and two troughs.

### Global metabolic and metabolite-specific responses to HCO_3_^−^ treatment

**Figure 5:**
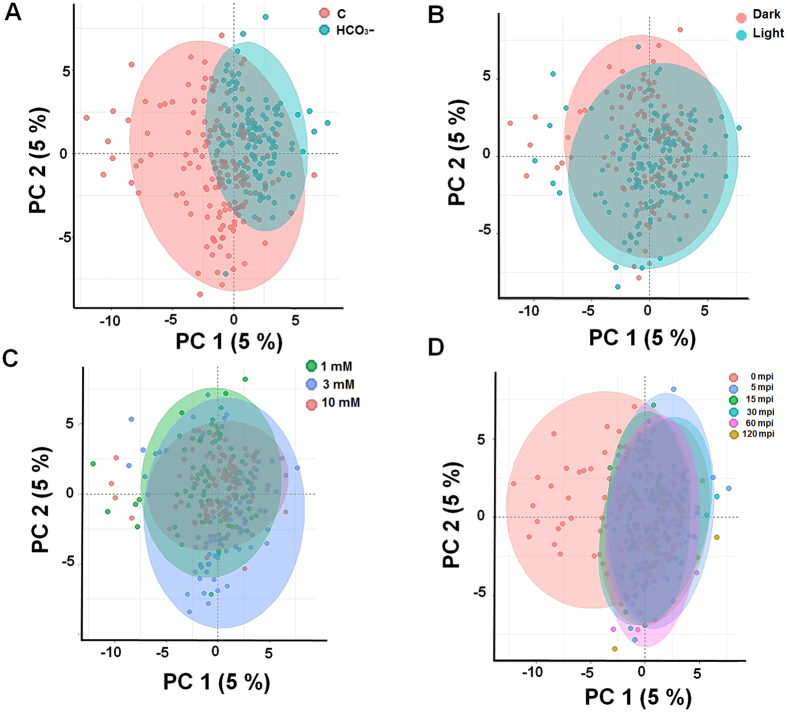
Orthogonal partial least square discriminant (OPLS-DA) analysis of metabolites. Alterations in *A. thaliana* suspension cells showed the effect of HCO_3_^−^. (**A**) treatment, (**B**) light conditions, (**C**) concentration, and (**D**) time-course upon HCO_3_^−^ treatment. OPLS-DA was performed using four replicates data of relative metabolite abundance in samples at 0, 5, 15, 30, 60, 120 min, and the generated PC1 and PC2 were plotted.

**Figure 6:**
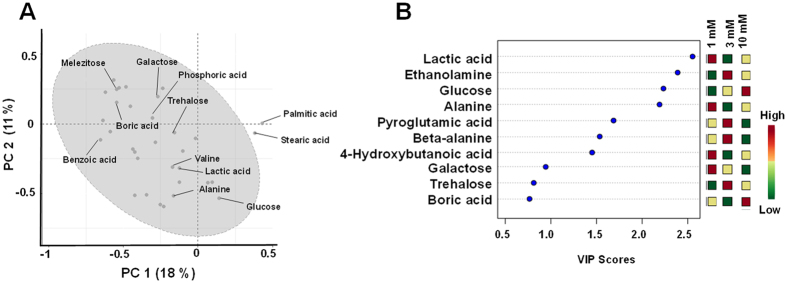
Principal component analysis of the GC-MS-based metabolites. (**A**) Loading plot displaying the contribution of individual metabolites; (**B**) Variable importance in projections (VIP) scores of top 10 metabolites obtained from the PLS-DA analysis.

In OPLS-DA, the supervised multivariate linear models revealed grouped and differential responses of the suspension cells to HCO_3_^−^ with an interpretable visualization in addition to providing the important metabolites, which helped in differentiating the various treatments (groups). Upon OPLS-DA evaluation, the resulting score plots indicated great separation of control and HCO_3_^−^ treatment (Fig. 5A), slight separation between dark and light-treated conditions (Fig. 5B), 3 mM standing out of the three concentrations (Fig. 5C), and a clear separation at 0 time point controls and the other time-points (Fig. 5D). In addition, flavonoids such as neohesperidin, isoliquiritigenin, and luteolin showed treatment and light-dependent changes in the time-course study (Fig. 5A–D). GC-MS based profiling of primary metabolites revealed the contribution of individual metabolites in the loading plots, i.e., sugars (glucose, galactose, trehalose and melezitose), amino acids (Val, Ala, Gly), and fatty acids (stearic and palmitic acid) among others (Fig. 6A). OPLS-DA analyses based variable importance in projections (VIP) scores indicated the roles of sugars (glucose, galactose, trehalose), amino acids (Ala, beta-Ala, Gly, pyroglutamic acid), lactic acid, ethanolamine, boric and phosphoric acids in the HCO_3_^−^ responsiveness (Fig. 6B). To further validate the models, we performed random permutation tests with the OPLS-DA model. In accordance with the OPLS-DA models, all the components and validation with 1000 random permutation tests generated intercepts with acceptable (>0.9) variance (R^2^) and the cross-validated variance (Q^2^) values (>0.85).

## Discussion

Increases in atmospheric CO_2_ concentrations provide additional resources for photosynthesis, which leads to high accumulation of photosynthate^10^,^23^. Carbonic anhydrase (CA) converts CO_2_ into HCO_3_^−^ for incorporation into cellular metabolism and acts as an upstream regulator of CO_2_^24^. CA catalyzes the reaction CO_2_ + H_2_O ↔ HCO_3_^−^ + H^+^ in plants leading to accumulated HCO_3_^−^, where rapid equilibration leads to increased concentration of CO_2_ around RuBisCO. In addition, the fundamental function of PEPCase is to catalyze the conversion of HCO_3_^−^ and phosphoenolpyruvate (PEP) to oxaloacetate. In dicots, PEPCases are activated by glucose 6-phosphate (G6P) and inhibited by malate or Asp^25^. Furthermore, the dark fixation of CO_2_/HCO_3_^−^ is important for plant growth^26^. Our results showed wide-spread shifts in cellular metabolism at all four levels, i.e., in response to treatment (control and HCO_3_^−^) (Fig. 6A), light conditions (dark and light) (Fig. 1A), concentrations (1, 3, and 10 mM) (Fig. 1B) and time-course (0, 5, 15, 30, 60, 120 min) (Table 1). We observed large-scale HCO_3_^−^ concentration-dependent metabolomic changes in the photomixotrophic *A. thaliana* suspension cells. In addition, transcriptomic reprogramming in response to modulation of light can occur in a matter of seconds in *A. thaliana*^13^. We observed more synchronized and comparable responses at 3 mM HCO_3_^−^ as compared to low (1 mM) or high (10 Mm) HCO_3_^−^, possibly because those concentrations are either too low to elicit a cellular response or too high that they trigger stress responses and suppress other metabolic processes. Evidently, saturated photosynthesis takes place in soybean leaf mesophyll cells under light at 10 mM HCO_3_^−^ 27. Moreover, photosynthetic activities in photosystem II (PSII) are known to increase when treated with 1 mM HCO_3_^−^, but not when treated with 12 mM HCO_3_^−^, indicating that higher concentration may not be activating PSII, but only playing a protective role against excessive damage^28^. In addition, dose-dependency of HCO_3_^−^ (increments from 2 mM to 5 mM HCO_3_^−^) in inducing malate and citrate synthesis in plant cells is also known^29^. Thus, in this study the observed metabolomic responses not only reflect HCO_3_^−^ dependent responses but also species-specific responses.

Recently, microarray analyses conducted in moss (*P. patens*) gametophytes revealed that the expression levels of 814 genes were affected under elevated CO_2_, where most transcriptional reprogramming occurred in photosynthetic regulation, carbon metabolism, and stress responses^9^. In our metabolomic data sets, pyrimidine metabolism and folate metabolism showed unique responses to light and dark conditions, respectively, whereas flavonoid metabolism, glutathione, and alkaloid metabolism were enriched in both dark and light conditions. In fact, light induced phenylpropanoid metabolism and flavonoid biosynthesis in *A. thaliana* roots, as a high-irradiance response is well documented^30^. Increased folate metabolism in dark conditions indicates stress response as reiterated from the involvement of folate in seedling establishment of *A. thaliana* in dark^31^. Interestingly, lactic acid, a biomarker for concentration-dependent response to HCO_3_^−^, displayed the highest VIP score derived from PLSDA analyses. Lactic acid is a known indicator of hypoxia stress in maize roots^32^. Hence, lactic acid production is also indicative of anoxic and stressful conditions leading to anaerobic respiration in photosynthetic suspension cells in response to HCO_3_^−^. Similarly, induction of secondary metabolites, e.g., alkaloids by elevated CO_2_ is known in many alkaloid producing plants^33^. Furthermore, in cyanobacterial model *Cyanothece* sp. PCC 7822, proteomics and transcriptomics indicated that proteins for nitrogenase and the pentose phosphate pathway were increased in the dark, whereas glycolysis and TCA cycle were more prominent in the light^34^. Furthermore, Glu, a proteogenic amino acid and indicator of protein abundance in plant cells displayed HCO_3_^−^ concentration dependent accumulation patterns. We observed the levels of benzoic acid, a phenylpropanoid metabolism derivative, accumulated only in the dark, where its levels were depleted by HCO_3_^−^ under light (Supplementary Table S4). Thus, not only does HCO_3_^−^ perturbs light-dependent secondary metabolism, but also results in shifts in respiration, transcription (light-induced changes in pyrimidine metabolism and concentration-dependent changes in purine metabolism), amino acid (Ala and pyroglutamic acid with VIP > 1), sugar (glucose, galactose, trehalose with VIP > 1) and folate metabolism.

Significant changes in glutathione metabolism under light (Fig. 2A) at both 1 mM and 10 mM HCO_3_^−^ (Fig. 2B) indicate the involvement of redox regulation in presence of HCO_3_^−^ (Table 1). Diurnal and light-mediated regulation of sulfur and glutathione metabolism in *A. thaliana* is known^35^. Upon HCO_3_^−^ treatment, glutathione metabolism, an integral part of redox regulation showed changes in both light and concentration-dependent manner. Increased oxidative stress in C3 plants^36^ and decreased ascorbate by high CO_2_^11^ further supports redox associated changes upon HCO_3_^−^ treatment. Changes in taurine and flavonoid metabolism (Fig. 5, Table 1) with time further substantiate the role of antioxidants in amelioration of elevated HCO_3_^−^ induced oxidative responses. In a recent study, up-regulation of genes encoding enzymes involved in oxidative signaling and ROS scavenging such as glutathione-S-transferase, peroxidases, catalases, Cys and thioredoxin-pathway were reported under elevated CO_2_^9^. Thus, elevated CO_2_ can enhance maintenance of the redox potential due to elevated rates of CO_2_ assimilation and electron transport, as well as low photorespiration^37^. In a nutshell, cellular redox regulation is intricately associated with HCO_3_^−^ responses, and eventually with elevated CO_2_ conditions. In addition, using HCO_3_^−^ feeding/enrichment as an alternative to elevated CO_2_, we show the usefulness of this system in *A. thaliana* suspension cell cultures which can be extended to other cell-types and plant cultures.

Increased levels of metabolites from glycolysis/gluconeogenesis, galactose, starch and sucrose metabolism along the time-course (Supplementary Table S3) are consistent with previous results of CO_2_ induced accumulation of sugars and starch^38^. Although maintenance of a C/N balance in plants is complex and mediated by multiple mechanisms^39^, we observed metabolic flow in favor of primary metabolism with increased HCO_3_^−^. In contrast, metabolites showing decreases throughout the time-course belonged to fatty acid, vitamin B6, pantothenate metabolism among others (*p* < 0.1). Phe is involved in biosynthesis of secondary metabolites (phenylpropanoids and flavonoids). Its increase possibly indicated increased pathway fluxes to generate more defense-related metabolites. Furthermore, elevated CO_2_ is known to associate with increased secondary metabolites, e.g., phenolics, flavonoids, alkaloids and terpenoids^38^. Increased secondary metabolism is a characteristic of light signal, which induce systems-wide changes in rice transcriptome^15^. Thus, HCO_3_^−^ not only regulates primary metabolism, but also modulates secondary metabolism in plant cells indicating a genome-wide metabolic shifts in light-, concentration-, and time-dependent manner.

As time-resolved single-cell type metabolomic studies are rare, the concentration, light, and time-dependent HCO_3_^−^ induced metabolomic changes reported here in *A. thaliana* suspension cells would help us understand CO_2_ responsive metabolic changes. Typical cellular HCO_3_^−^ induced responses are characterized by significant changes in flavonoid, glutathione, and Phe metabolism in light- and concentration-dependent manner, indicating functional and temporal behavior in the cells. Validation of these regulated metabolic pathways by means of traditional and omics approaches is essential for a systems biology perspective on HCO_3_^−^ response. The results of this study have shown the utility of metabolomics tools towards improved understanding of the plant CO_2_ sensing, utilization, and response mechanisms under light and dark conditions.

## Materials and Methods

### Plant materials and culture conditions

*A. thaliana* var. Landsberg erecta (LER) suspension cell cultures were obtained from Dr. Joshua Heazlewood (University of Western Australia, Australia) and maintained in 250 ml flasks under illumination of 130 μmol m^−2^ s^−1^ on an orbital shaker with constant shaking at 120 rpm, 22 °C. They were sub-cultured weekly by tenfold dilution into fresh Murashige and Skoog (MS) medium^40^ supplemented with 3% sucrose, kinetin (0.05 mg/L) and α-naphthalene acetic acid (NAA) (0.5 mg/L).

### HCO_3_^−^ treatment of suspension cells

The suspension cells were treated with NaHCO_3_ at a final concentration of 1, 3, or 10 mM. For controls, NaNO_3_ (NO_3_^−^ is a macronutrient in MS media) was added in same concentration to nullify the effect of Na^+^ during the treatment. Furthermore, as HCO_3_^−^ induced significant pH changes of the MS media (pH 5.8), we added 50 mM (for 1 mM and 3 mM HCO_3_^−^) and 100 mM (for 10 mM HCO_3_^−^) MES buffer (pH 5.8) to the MS media, and the pH-stabilized MS media were used for both control and HCO_3_^−^ treatments. In addition, the buffered MS media were sonicated to remove atmospheric gases prior to addition of HCO_3_^−^. Cells were incubated at 25 °C for 0, 5, 15, 30, 60, and 120 min on a shaker, and four replicates (n = 4) were generated for each data point after treatment with HCO_3_^−^ (treatment) and control. After treatment, 100 ml cells at a concentration of 3 × 10^6^ ml^−1^ were filtered using filter paper mounted on funnels, quickly blotted dry using Kim wipes and immediately snap-frozen in liquid nitrogen and stored in a −80 °C freezer until metabolite extraction.

### Metabolite sample preparation and analysis

Authentic standard metabolites, listed in Supplementary Table S1 were obtained from Sigma-Aldrich (St. Louis, MO, USA) and their stocks were stored in −80 °C. Serial dilutions of the standards were tested over a range of 10 pmol μL^−1^ to 1 nmol μL^−1^ for the linearity of detection in the mass spectrometer. Metabolite extractions from 20 mg of freeze dried suspension cells were performed following Fiehn *et al*.^41^. During extraction internal standards were added, i.e., 33 pmol lidocaine (positive mode), 210 pmol camphor-10-sulfonic acid and 100 pmol ribitol (negative mode) to aid in retention time correction and peak abundance quantification. Extracts were dissolved in 100 μL ddH_2_O and aliquoted for HPLC (MRM)-MS and GC-MS analysis.

### Targeted profiling by HPLC-MRM-MS

The targeted metabolite profiling of the samples were performed using HPLC-MRM-MS as described^16^. An Agilent 1100 HPLC system (Agilent Technologies, Santa Clara, CA, USA) was used with an autosampler (Agilent, Santa Clara, CA, USA) to inject the samples. A C18-reverse phase analytical column (Gemini 5 *μ*; 150 × 2.0 mm, Phenomenex, Torrance, CA, USA) with the following gradient program helped separation of the compounds using two solvents i.e., 0.1% formic acid in H_2_O (solvent A) and 0.1% formic acid in acetonitrile (solvent B) for 1:99, v/v, at 0 min; 1:99, v/v, at 0.2 min; 99.5:0.5, v/v at 31 min; 99.5:0.5, v/v at 34 min; 1:99, v/v, at 34.2 min; 1:99 at 60 min at room temperature at a flow rate of 500 μL/min. The HPLC was coupled to a hybrid triple quadrupole-ion trap 4000 Q-TRAP system equipped with a TurboIonSpray (TIS) interface (AB Sciex Inc., Foster City, CA, USA). The electrospray ionization (ESI) parameters were as previously described^16^, while the optimized MRM assay parameters are in Supplementary Table S1. QQQ scans were acquired as MRM experiments, where we monitored 59 (5.8 min), 40 (9.9 min), 45 (7.9 min), 31 (12.5 min), and 18 (23.8 min) MRM transitions over five periods (segments) in positive ionization, and 66 and 11 MRM transitions for two periods in negative ionization^42^. Each MRM transition was obtained with a dwell times specific for compounds, and a total cycle time was 1–1.5 s to ensure at least 10 data points across the peak. Only water served as blank solutions, while ddH_2_O was injected during washes. Pooled samples from the entire study served as quality control for monitoring the chromatography and MS conditions. Data imported using Analyst^TM^ software version 1.5.1 (AB Sciex, Foster City, CA, USA) while peak areas were integrated and quantified using the IntelliQuan algorithm of the MultiQuant^TM^ software version 2.1 (AB Sciex, Foster City, CA, USA).

### Profiling of metabolomes by GC-MS

GC-MS was performed following Lisec *et al*.^43^, where 20 μl of aliquoted extracts were dried and were sequentially derivatised with 100 μl of 20 mg ml^−1^ methoxyamine hydrochloride (MeOX) in pyridine, followed by shaking at 55 °C for 90 min, then overnight incubation, which was followed by trimethylsilylation by adding 100 μl of *N*-methyl-*N*-trimethylsilyl-trifluoroacetamide (MSTFA) to the mixture and incubation for 30 min at 37 °C, 30 min at 60 °C, and finally for 120 min at 100 °C to obtain optimized derivatization Samples were injected at a split ratio of 1:25 (250 °C) with a He carrier gas flow of 2 ml/min in a GC-MS system consisting of an autosampler, a TRACE^TM^ 1310 gas chromatograph (Thermo Scientific, San Jose, CA, USA) in-line with a TSQ8000 Triple Quadrupole mass spectrometer (Thermo Scientific, San Jose, CA, USA) equipped with an electron ionization (EI) source. A TR-5MS capillary column (30 m × 0.25 mm × 0.25 μm, Thermo Scientific, Waltham, MA) was used and the temperature program was essentially as described^16^. A standard n-alkane mixture (C8-C40) was injected at the beginning and end of the analysis for tentative identification and to monitor any shifts in retention indices (RI). The GC-MS data obtained from Xcalibur™ software version 2.0 (Thermo Finnigan, Austin, TX, USA) in raw format were aligned and processed as described^44^. Filtered raw GC-MS data sets comprised of data from four biological replicates (n = 4) and 29 curated analytes with less than 10% missing values, which were imputed using 1/3^rd^ of minimum values in each sample. Metabolite identification and quantification were performed as described^16^ using MSRI spectral libraries of Golm Metabolome Database available from Max-Planck-Institute for Plant Physiology, Golm, Germany (http://csbdb.mpimp-golm.mpg.de/csbdb/gmd/gmd.html)^45^, Automated Mass Spectral Deconvolution and Identification System (AMDIS, National Institute of Standards and Technology, USA) and NIST Mass Spectral Reference Library (NIST11/2011; National Institute of Standards and Technology, USA) library.

### Statistical analysis

The individual MS data were assembled into a concatenated data set into a single. csv sheet in Microsoft Excel (Microsoft Corp., Seattle, WA, USA) before statistical analyses using statistical software R (Version 2.9.1, R Development Core Team 2007, https://www.r-project.org/)^46^ Logarithmic and median transformations of metabolite data were performed using DeviumWeb^47^ and MetaboAnalyst^48^, and data were transformed, scaled, missing values imputed, and outlier removed before proceeding to uni- and multi-variate statistical analysis of the data. Raw processed data are available in Supplementary Table S2. ANOVA was performed using DeviumWeb^47^. *p*-values were adjusted by Benjamini-Hochberg correction (BH)^49^. Probability level, α was set to 0.05, adjusted for multiple hypotheses testing using BH to allow for a maximum 5% probability (q = 0.05) in false positive detection (FDR). Metabolite fold changes with cut-offs of 1.2 and 0.8, and significant changes (*p* < 0.05) were calculated (Supplementary Table S3). Principal component analysis (PCA) and orthogonal partial least square discriminant analysis (OPLS-DA) were performed using DeviumWeb^47^, where output consisted of score plots to visualize the contrast between different samples and loading plots to explain the cluster separation. The data files were scaled with unit variance without any transformations.

### Pathway enrichment analysis and clustering analysis

Pathway enrichment, over-representation, and mapping were performed using functionalities available at MBRole (http://csbg.cnb.csic.es/mbrole/), MSEA (http://www.msea.ca/MSEA/) and MetPA (http://metpa.metabolomics.ca/MetPA/) while ID conversions were performed at Chemical Translation Service (CTS: http://cts.fiehnlab.ucdavis.edu/conversion/batch) (Supplementary Table S1). Short Time series Expression Miner (STEM)^50^ was used as a Java implementation with a graphical user interface available at http://www.cs.cmu.edu/~jernst/st/ for clustering the metabolite accumulation patterns according to time points. Hierarchical clustering analysis (HCA) using average linkage clustering was performed on Euclidean distances using PermutMatrix^51^.

## Additional Information

**How to cite this article**: Misra, B. B. *et al*. Metabolomic Responses of Arabidopsis Suspension Cells to Bicarbonate under Light and Dark Conditions. *Sci. Rep.* **6**, 35778; doi: 10.1038/srep35778 (2016).

## Supplementary Material

Supplementary Information

## Figures and Tables

**Table 1:** Within subject-ANOVA showing enriched pathways based on significantly changed metabolites for time, light, treatment, concentrations, and their interactions.

Pathways	Total Metabolites	Hits	P-value
**Time**			
Taurine and hypotaurine metabolism	5	2	0.04117
**Light**			
Flavone and flavonol biosynthesis	9	4	0.003184
Flavonoid biosynthesis	43	9	0.00434
Glutathione metabolism	26	6	0.012087
Phenylalanine metabolism	8	3	0.019084
**Treatment**			
Flavone and flavonol biosynthesis	9	4	7.77E-04
Taurine and hypotaurine metabolism	5	2	0.025468
**Concentration**			
Flavone and flavonol biosynthesis	9	5	0.004267
Flavonoid biosynthesis	43	11	0.032193
Glutathione metabolism	26	7	0.063731
beta-Alanine metabolism	12	4	0.07746
Pyrimidine metabolism	38	9	0.077932
Purine metabolism	61	13	0.079759
Phenylalanine metabolism	8	3	0.09171
Tyrosine metabolism	18	5	0.099267
**Interactions**			
Glyoxylate and dicarboxylate metabolism	17	2	0.035261
Alanine, aspartate and glutamate metabolism	22	2	0.056748
Taurine and hypotaurine metabolism	5	1	0.086283
