# Supplementary material for: Metabolomic Responses of Arabidopsis Suspension Cells to Bicarbonate under Light and Dark Conditions
Source: Sci Rep. 2016 Oct 20;6:35778. doi: 10.1038/srep35778 (PMC5071901; doi:10.1038/srep35778)
Supplement: Supplementary Information [file srep35778-s1.pdf]

Metabolomic Responses of Arabidopsis Suspension Cells to Bicarbonate under Light and Dark Conditions

Biswapriya B. Misra[1], Zepeng Yin[1,2], Sisi Geng[1], Evaldo de Armas[3], Sixue Chen[1,4*]

[1]Department of Biology, Genetics Institute, Plant Molecular and Cellular Biology Program, University of Florida, Gainesville, FL 32610, USA

[2]Alkali Soil Natural Environmental Science Center, Northeast Forestry University, Key Laboratory of Saline-alkali Vegetation Ecology Restoration in Oil Field, Ministry of Education, Harbin 150040, China

[3]Training Institute, Thermo Fisher Scientific, 1400 North point Parkway, Ste 10., West Palm Beach, FL 33407, USA

[4]Interdisciplinary Center for Biotechnology Research, University of Florida, Gainesville, FL 32610, USA

***** Correspondence: schen@ufl.edu

Supplementary Information. Additional Supporting Information may be found in the online version of this article at the publisher's website.

Figure S1: Reference KEGG metabolic pathway overview showing the quantified metabolites. Black dots indicate the identified and quantified metabolites using HPLC-MRM-MS and GC-MS in this investigation.

Table S1. Metabolites used in the time-course profiling study for the targeted analysis by HPLC-MRM-MS/MS. KEGG: Kyoto Encyclopedia of Genes and Genomes, HMDB: Human Metabolome Database, InChI Key: IUPAC International Chemical Identifier, CAS: Chemical Abstracts Service (CAS) number, ESI: Electron Spray Ionization, Q1:Precursor Ion, Q2: Daughter Ion (Transition), DP: Declustering Potential, CE: Collision Energy, CXP: Cell Exit Potential (10 and -10 for positive and negative modes for the 4000 QTRAP (ABSciex) used.

Table S2. Normalized and scaled peak areas (as relative abundances) of metabolites quantified using HPLC-MRM-MS/MS analyses at 0, 5, 15, 30, 60, and 120 mpi HCO_3^- treatment (T) and control (C) for *A. thaliana* suspension cells under light (L) and dark (D) conditions at three different concentrations (1, 3, and 10 mM) (n=4).

Table S3. Fold changes (1.2 and 0.8 as cut-offs) and significantly changed metabolites ($P < 0.1$) detected using the HPLC-MRM-MS/MS platform at 0, 5, 15, 30, 60, and 120 mpi HCO_3^- treatment (T) and control (C) for *A. thaliana* suspension cells under light (L) and dark (D) conditions at three different concentrations (1, 3, and 10 mM) as inferred from ANOVA.

Table S4. Fold changes (1.2 and 0.8 as cut-offs) and significantly changed metabolites ($P < 0.1$) detected by GC-MS platform at 0, 5, 15, 30, 60, and 120 mpi HCO_3^- treatment (T) and control

(C) for *A. thaliana* suspension cells under light (L) and dark (D) conditions at three different concentrations (1, 3, and 10 mM) as inferred from ANOVA.

Table S5. Significantly changed metabolites ($P < 0.05$) at 0, 5, 15, 30, 60, and 120 mpi HCO_3^- treatment (T) and control (C) for *A. thaliana* suspension cells under light (L) and dark (D) conditions at three different concentrations (1, 3, and 10 mM) and their interactions as inferred from within subject ANOVA models.

Table S6. Pathways enrichment of STEM analysis patterns of metabolite changes across light conditions (light and dark) and HCO_3^- concentrations (1, 3, and 10 mM). Numbers indicate the number of metabolites associated with each model.

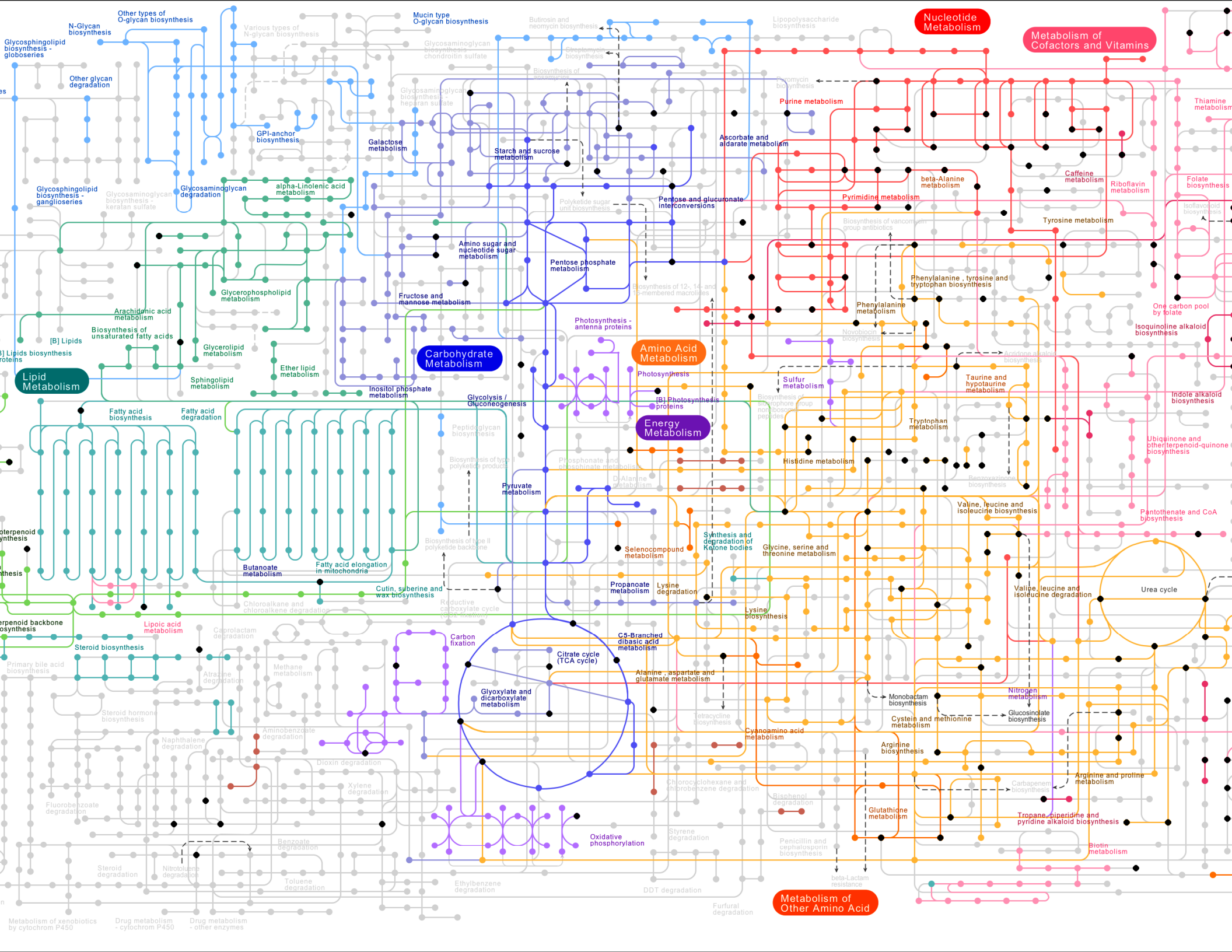
Nucleotide Metabolism
Metabolism of Cofactors and Vitamins
Carbohydrate Metabolism
Amino Acid Metabolism
Lipid Metabolism
Energy Metabolism
Metabolism of Other Amino Acid
Other types of O-glycan biosynthesis
N-Glycan biosynthesis
Mucin type O-glycan biosynthesis
Various types of N-glycan biosynthesis
Glycosphingolipid biosynthesis - globoseries
Other glycan degradation
GPI-anchor biosynthesis
Galactose metabolism
Starch and sucrose metabolism
Ascorbate and aldarate metabolism
Purine metabolism
Thiamine metabolism
Glycosphingolipid biosynthesis - ganglioseries
Glycosaminoglycan degradation
alpha-Linolenic acid metabolism
Pentose and glucuronate interconversions
Caffeine metabolism
Riboflavin metabolism
Folate biosynthesis
beta-Alanine metabolism
Pyrimidine metabolism
Tyrosine metabolism
Amino sugar and nucleotide sugar metabolism
Pentose phosphate metabolism
Glycerophospholipid metabolism
Fructose and mannose metabolism
Phenylalanine , tyrosine and tryptophan biosynthesis
Phenylalanine metabolism
One carbon pool by folate
Isoquinoline alkaloid biosynthesis
Arachidonic acid metabolism
Biosynthesis of unsaturated fatty acids
[B] Lipids
Glycerolipid metabolism
Photosynthesis - antenna proteins
Photosynthesis
[B] Photosynthesis proteins
Sulfur metabolism
Taurine and hypotaurine metabolism
Indole alkaloid biosynthesis
Ether lipid metabolism
Sphingolipid metabolism
Inositol phosphate metabolism
Glycolysis / Gluconeogenesis
Fatty acid biosynthesis
Fatty acid degradation
Tryptophan metabolism
Ubiquinone and other terpenoid-quinone biosynthesis
Histidine metabolism
Pyruvate metabolism
Valine, leucine and isoleucine biosynthesis
Pantothenate and CoA biosynthesis
Synthesis and degradation of Ketone bodies
Glycine, serine and threonine metabolism
Selenocompound metabolism
Butanoate metabolism
Fatty acid elongation in mitochondria
Cutin, suberine and wax biosynthesis
Propanoate metabolism
Lysine degradation
Valine, leucine and isoleucine degradation
Urea cycle
Lysine biosynthesis
Lipoic acid metabolism
Steroid biosynthesis
Carbon fixation
C5-Branched dibasic acid metabolism
Citrate cycle (TCA cycle)
Alanine , aspartate and glutamate metabolism
Glyoxylate and dicarboxylate metabolism
Monobactam biosynthesis
Nitrogen metabolism
Cysteine and methionine metabolism
Glucosinolate biosynthesis
Cyanoamino acid metabolism
Arginine biosynthesis
Arginine and proline metabolism
Glutathione metabolism
Tropane, piperidine and pyridine alkaloid biosynthesis
Biotin metabolism
Oxidative phosphorylation
beta-Lactam resistance
Metabolism of xenobiotics by cytochrom P450
Drug metabolism - cytochrom P450
Drug metabolism - other enzymes

Table S1. Metabolites used in the time-course profiling study for the targeted analysis by HPLC-MRM-MS/MS. KEGG: Kyoto Encyclopedia of Genes and Genomes, HMDB: Human Metabolome Database, InChI Key: IUPAC International Chemical Identifier, CAS: Chemical Abstracts Service (CAS) number, ESI: Electron Spray Ioni

Compounds	Forumula	MW (Da)	KEGG ID	HMDB ID	InChI Key	CAS Numbers	Retention Ti	Q1	Q3	ESI Mode	DP	CE
α-Ketoisovaleric acid	$C_5H_8O_3$	116.11522	C00141	HMDB00019	QHKABHOOEWYVLI-UHFFFAOYSA-N	-	51.9	115.1	55	-	-63	-47
(6R)-5,6,7,8-Tetrahydrobiopterin	$C_9H_{15}N_5O_3$	241.2471	C15522	HMDB02281	FNKQXYHWGSIFBK-BYAPIUGTSA-N	-	17.1	351.96	220.2	+	22	25
1,3-Diaminopropan	$NH_2(CH_2)_3NH_2$	74.12	C00986	HMDB00002	XFNJVJPLKCPIBV-UHFFFAOYSA-N	109-76-2	4.37	75.1	58.1	+	32	14
1,7-Dimethylxanthine	$C_7H_8N_4O_2$	180.16402	C13747	HMDB01860	QUNWUDVFRNGTCO-UHFFFAOYSA-N	611-59-6	16.4	181.1	99.1	+	70	35
1-Aminocyclopropane-1-carboxylic acid	$C_4H_7NO_2$	101.10388	C01234	-	PAJPWUMXBYXFCZ-UHFFFAOYSA-N	22059-21-8	4.65	101.9	56.1	+	35	17
1-Kestose	$C_{18}H_{32}O_{16}$	504.43708	C03661	HMDB11729	VAWYEUIPHLMNNF-OESPXIITSA-N	470-69-9	16.47	503.1	89.1	-	-62	-49
1-Methylguanidine	$C_2H_7N_3$	73.09708	C02294	HMDB01522	CHJJGSNFBQVOTG-UHFFFAOYSA-N	-	4.5	74.063	57	+	47	11
1-Methylhistamine	$C_6H_{11}N_3$	125.17164	C05127	HMDB00898	FHQDWPCFSJMNCT-UHFFFAOYSA-N	-	4.91	126.1	109.1	+	37	21
1-Methylhistidine	$C_7H_{11}N_3O_2$	169.18114	C01152	HMDB00001	BRMWTNUJHUMWMS-LURJTMIESA-N	-	4.9	170.18	124	+	38	23
1-Methylnicotinamide	$C_7H_9N_2O^+$	137.15916	C02918	HMDB00699	LDHMAVIPBRSVRG-UHFFFAOYSA-O	-	4.28	138.3	95.1	+	94	36
1-Monooctadecanoyl-rac-glycerol	$C_{21}H_{42}O_4$	358.555786	D01947	-	VBICKXHEKHSIBG-UHFFFAOYSA-N	123-94-4	59.8	357.5	283.3	-	-35	-28
1-Octacosanol	$C_{28}H_{58}O$	410.75952	C08387	HMDB02348	CNNRPFQICPFDPO-UHFFFAOYSA-N	557-61-9	7.36	387.1	171	+	14	37
1-Oleoyl-rac-glycerol	$C_{21}H_{40}O_4$	356.5399	C04230	HMDB02815	RZRNAYUHWVFMIP-KTKRTIGZSA-N	-	57.97	325.1	183	-	-38	-56
1-Tetracosanol	$CH_3(CH_2)_{23}OH$	354.65	_	-	TYWMIZZBOVGFOV-UHFFFAOYSA-N	506-51-4	57.97	339.1	182.9	-	-39	-63
2,3-Dihydroxybenzoic acid	$C_7H_6O_4$	154.12014	C00196	HMDB00397	GLDQAMYCGOIJDV-UHFFFAOYSA-N	303-38-8	35.25	155.04	137.04	+	44	22
2,3-Pyridinedicarboxylic acid	$C_7H_5NO_4$	167.1189	C03722	HMDB00232	GJAWHXHKYYXBSV-UHFFFAOYSA-N	89-00-9	18.43	168.12	150	+	21	16
22R,23R,24R-2α,3α,22,23-Tetrahydroxy-B-homo-7-oxa-5α-ergostan-6-one	$C_{28}H_{48}O_6$	480.68	C11049	-	IXVMHGVQKLDRKH-QHBHMFGVSA-N	78821-43-9	51	481.2	445.4	+	40	20
2-aminoethylphosphonic acid	$C_2H_8NO_3P$	125.063582	C03557	HMDB11747	QQVDJLLNRSOCEL-UHFFFAOYSA-N	2041-14-7	4.66	125.8	96.9	+	56	31
2-Deoxy inosine-5-triphosphate	$C_{10}H_{15}N_4O_{13}P_3$	492.166386	C01345	HMDB03537	UFJPAQSLHAGEBL-RRKCRQDMSA-N	-	19.9	331.1	135	-	-47	-45
2-Deoxy uridine	$C_9H_{12}N_2O_5$	228.20198	C00526	HMDB00012	MXHRCPNRJAMMIM-SHYZEUOFSA-N	-	17.73	229.2	113.1	+	32	45
2-Deoxyadenosine	$C_{10}H_{13}N_5O_3$	251.24192	C00559	HMDB00101	OLXZPDWKRNYJJZ-RRKCRQDMSA-N	-	17.07	252.1	136.1	+	43	25
2-Deoxyadenosine-5-monophosphate	$C_{10}H_{12}N_5O_6P^{-2}$	329.205942	C00360	HMDB00905	KHWCHTKSEGGWEX-RRKCRQDMSA-N	-	16.9	332.1	136.1	+	47	26
2-Deoxycytidine-5-diphosphate	$C_9H_{12}N_3O_{10}P_2^{-3}$	384.153204	C00705	HMDB01245	FTDHDKPUHBLBTL-SHYZEUOFSA-N	-	11.3	386.2	158.7	-	-38	-67
2-Deoxyguanosine	$C_{10}H_{13}N_5O_4$	267.24132	C00330	HMDB00085	YKBGVTZYEHREMT-KVQBGUIXSA-N	-	18.07	268.2	152.1	+	43	36
2-Deoxyguanosine-5-monophosphate	$C_{10}H_{13}N_5O_7P$	346.213282	C00362	HMDB01044	LTFMZDNNPPEQNG-KVQBGUIXSA-N	-	17.33	348.22	136	+	44	35
2-Deoxyinosine	$C_{10}H_{12}N_4O_4$	252.22668	C05512	HMDB00071	VGONTNSXDCQUGY-RRKCRQDMSA-N	890-38-0	18.4	251.1	134.9	-	-63	-31
2-Deoxyribose-5-phosphate	$C_5H_{11}O_7P$	214.110402	C00673	HMDB01031	KKZFLSZAWCYPOC-PYHARJCCSA-N	-	9.13	213.1	96.8	-	-43	-59
2-Deoxyuridine-5-monophosphate	$C_9H_{13}N_2O_8P$	308.181882	C00365	HMDB01409	JSRLJPSBLDHEIO-SHYZEUOFSA-N	-	33.8	353.2	125	+	150	36
2-Hydroxybenzaldehyde	$C_7H_6O_2$	122.12134	C00755	HMDB12308	SMQUZDBALVYZAC-UHFFFAOYSA-N	121-33-5	31.8	121.1	77	-	-25	-13
2-Hydroxycinnamic acid	$C_9H_8O_3$	164.15802	C03549	HMDB02641	PMOWTIHVNWZYFI-AATRIKPKSA-N	-	30.03	163.1	119.1	-	-50	-20
2-Hydroxyiisobutyric acid	$C_4H_8O_3$	104.10452	C01188	HMDB00023	BWLBGMIXKSTLSX-UHFFFAOYSA-N	1910-47-0	16.73	102.9	56.9	-	-86	-15
2-Hydroxyphenylacetic acid	$C_8H_8O_3$	152.14732	C05852	HMDB00669	IWYDHOAUDWTVEP-UHFFFAOYSA-N	614-75-5	27.07	151	107.1	-	-21	-20
2-Hydroxypyridine	C_5H_5NO	95.0993	C01020	HMDB02658	UBQKCCHYAOITMY-UHFFFAOYSA-N	5006-66-6	17.47	95.5	78	+	93	31
2-Isopropylmalic acid	$C_7H_{12}O_5$	176.16718	C02504	HMDB00402	BITYXLXUCSKTJS-ZETCQYMHSA-N	-	23.7	175	114.9	-	-29	-23
2-Methoxycinnamic acid	$C_{10}H_{10}O_3$	178.1846	C10470	HMDB00955	FEGVSPGUHMGGBO-SREVYHEPSA-N	537-73-5	34.9	179.18	161	+	30	9
2-Methoxycinnamic acid	$C_{10}H_{10}O_3$	178.1846	C10470	HMDB00955	AFDXODALSZRGIH-QPJJXVBHSA-N	537-73-5	34.9	179.18	161	+	30	9
3, 4-Dihydroxy Phenyl acetic acid	$C_8H_8O_4$	168.14672	C01161	HMDB01336	CFFZDZCDUFSOFZ-UHFFFAOYSA-N	102-32-9	32.8	167	122.9	-	-24	-31
3, 4-Dihydroxy phenyl alanine	$C_9H_{11}NO_4$	197.18794	C07194	HMDB11754	WTDRDQBEARUVNC-UHFFFAOYSA-N	1339-75-9	9.66	198.1	107	+	52	41
3,4-Hydroxyphenylpropionic acid	$C_{14}H_{13}F_5O_4$	340.242636	C11457	HMDB00375	PZSJJAIUXKEYJA-UHFFFAOYSA-N	-	27.3	167.2	106.9	+	20	47
3,5-Dihydroxy-4,6,7-trimethoxyflavone	$C_{18}H_{16}O_7$	344.32	_	-	KLAOKWJLUQKWIF-UHFFFAOYSA-N	855-96-9	37.4	345.2	284.2	+	30	45
3-dephospho CoA	$C_{21}H_{33}N_7O_{13}P_2S^{-2}$	685.538344	C00882	HMDB01373	KDTSHFARGAKYJN-IBOSZNHHSA-N	-	25.1	686.3	339.3	-	-65	-49
3-Hydroxy 4-methoxycinnamic acid	$C_{10}H_{10}O_4$	194.184	C10470	HMDB00955	QURCVMIEKCOAJU-HWKANZROSA-N	537-73-5	28.37	193	133	-	-49	-40
3-Hydroxyanthranilic acid	$C_7H_7NO_3$	153.13538	C00632	HMDB01476	WJXSWCUQABXPFS-UHFFFAOYSA-N	-	11.5	154.14	136	+	29	10
3-Isopropylmalic acid	$C_7H_{12}O_5$	176.16718	C04411	HMDB12156	RNQHMTFBUSSBJQ-CRCLSJGQSA-N	-	23.6	177.12	71.04	+	23	15
3-Methyl xanthine	$C_6H_6N_4O_2$	166.13744	C16357	HMDB01886	GMSNIKWWOQHZGF-UHFFFAOYSA-N	-	19.4	165	65.9	-	-63	-43
3-Methyladenine	$C_6H_7N_5$	149.15328	C00913	HMDB11600	FSASIHFSFGAIJM-UHFFFAOYSA-N	5142-23-4	6.54	150.15	109	+	62	24
3-Methyl-L-histidine	$C_7H_{11}N_3O_2$	169.18114	C01152	HMDB00479	JDHILDINMRGULE-LURJTMIESA-N	-	4.55	170.085	96	+	52	24
3-Ureidopropionic acid	$C_4H_8N_2O_3$	132.11792	C02642	HMDB00026	JSJWCHRYRHKBBW-UHFFFAOYSA-N	-	8.42	133.12	115	+	26	10
4-Hydroxyphenylacetic acid	$HOC_6H_4CH_2CO_2H$	152.15	C00642	HMDB00020	XQXPVVBIMDBYFF-UHFFFAOYSA-N	533-22-2	27.07	150.5	107	-	-31	-33
4- Nitrophenyl phosphate	$C_6H_6NO_6P$	219.088702	C03360	HMDB01300	XZKIHKMTEMTJQX-UHFFFAOYSA-N	-	48.6	371	355	+	77	26
4- Nitrophenyl phosphate	$C_6H_5NO_3$	139.1088	C00870	HMDB01232	BTJIUGUIPKRLHP-UHFFFAOYSA-N	100-02-7	48.6	371	355	+	77	26
4,2,4-Trihydroxychalcone	$C_{15}H_{12}O_4$	256.25	C08650	-	DXDRHHKMWQZJHT-FPYGCLRLSA-N	961-29-5	57.97	255.1	209.1	-	-44	-12
4,7-Dihydroxyflavanone	$C_{15}H_{12}O$	256.25338	C09827	-	FURUXTVZLHCCNA-AWEZNQCLSA-N	-	31.87	257.2	137.1	+	67	40
4-Hydroxy-3-methoxycinnamyl alcohol	$C_{10}H_{12}O_3$	180.20048	C01494	-	JMFRWRFFLBVWSI-NSCUHMNNSA-N	537-98-4	31.3	209.1	150.9	-	-70	-40
4-Hydroxybenzoic acid	$C_7H_6O_3$	138.12074	C00156	HMDB00500	FJKROLUGYXJWQN-UHFFFAOYSA-N	-	25.07	137.12	93	-	-53	-8
4-Hydroxyphenyl pyruvic acid	$C_9H_8O_4$	180.15742	C01179	HMDB00707	KKADPXVIOXHVKN-UHFFFAOYSA-N	156-39-8	17.8	178.6	107	-	-54	-13
4-Hydroxyphenyl-β-D-glucopyranoside	C12H16O7	272.25	C06186	-	BJRNKVDFDLYUGJ-RMPHRYRLSA-N	497-76-7	17.4	271	107.9	-	-55	-38
4-Imidazoleacrylic acd	$C_6H_6N_2O_2$	138.12404	C00785	HMDB00301	LOIYMIARKYCTBW-OWOJBTEDSA-N	-	7.07	138.5	93	+	63	31
5,6-Dihydrouracil	$C_4H_6N_2O_2$	114.10264	C00429	HMDB00076	OIVLITBTBDPEFK-UHFFFAOYSA-N	504-07-4	4.49	114.9	69.9	+	61	28
5-Aminovaleric acid	$C_5H_{11}NO_2$	117.14634	C00431	HMDB03355	JJMDCOVWQOJGCB-UHFFFAOYSA-N	660-88-8	4.67	117.9	83	+	77	27

5-Deoxy-5-methylthio-adenosine	$C_{11}H_{15}N_5O_3S$	297.33	C00170	-	WUUGFSXJNOTRMR-WOIOKPISSA-N	2457-80-9	20.1	298.1	136.1	+	62	27
5-Hydroxyindole-3-acetic acid	$C_{10}H_9NO_3$	191.18336	C05635	HMDB00763	DUUGKQCEGZLZNO-UHFFFAOYSA-N	-	6.55	190	143.4	-	-82	-33
5-methoxytryptamine	$C_{11}H_{14}N_2O$	190.24166	C05659	HMDB04095	JTEJPPKMYBDEMY-UHFFFAOYSA-N	608-07-1	29	191.1	163	+	16	54
5-Methylcytosine	$C_5H_7N_3O$	125.12858	C02376	HMDB02894	LRSASMSXMSNRBT-UHFFFAOYSA-N	554-01-8	5.05	125.7	109	+	69	44
6,7-Dihydroxycoumarin	$C_9H_6O_4$	178.14	C09263	-	ILEDWLMCKZNDJK-UHFFFAOYSA-N	305-01-1	31.07	177	133	-	-55	-15
6-Amino caproic acid	$C_6H_{13}NO_2$	131.17292	C02378	HMDB01901	SLXKOJJOQWFEFD-UHFFFAOYSA-N	93208-38-9	6.47	132.2	114	+	40	12
6-Furfurylaminopurine	$C_{10}H_9N_5O$	215.21136	C08272	HMDB12245	QANMHLXAZMSUEX-UHFFFAOYSA-N	525-79-1	21.87	216	81	+	30	40
6-Hydroxy flavone	$C_{15}H_{10}O_3$	238.2381	C14137	-	GPZYYYGYCRFPBU-UHFFFAOYSA-N	6665-83-4	34.83	237.2	101	-	-79	-47
6-Phosphogluconic acid	$C_6H_{13}O_{10}P$	276.135182	C00345	HMDB01316	BIRSGZKFKXLSJQ-SQOUGZDYSA-N	-	7.91	275.1	96.9	-	-47	-54
6-γ,γ-Dimethylallylaminopurine	$C_{10}H_{13}N_5$	203.24	C04083	-	HYVABZIGRDEKCD-UHFFFAOYSA-N	2365-40-4	22.8	202.1	131.9	-	-47	-46
7-Hydroxyflavone	$C_{15}H_{10}O_3$	238.24	C11264	-	MQGPSCMMNJKMHQ-UHFFFAOYSA-N	6665-86-7	34.83	237.2	90.9	-	-30	-45
8-nitroguanosine 3,5-cyclic monophosphate	$C_{10}H_{10}N_6O_9P$	412.2	-	-	-	-	30.2	388.5	195.2	-	-95	-32
Abscisic acid	$C_{15}H_{20}O_4$	264.3169	C06082	-	JLIDBLDQVAYHNE-YKALOCIXSA-N	-	31.2	265.08	247.08	+	26	8
Abscisic acid-beta-D-glucopyranosyl ester	$C_{21}H_{30}O_9$	426.4575	C15970	-	HLVPIMVSSMJFPS-VTEUUMMASA-N	-	6.21	427.2	247.1	+	30	17
Acetylcholine	$C_7H_{16}NO_2^+$	146.20744	C01996	HMDB00895	OIPILFWXSMYKGL-UHFFFAOYSA-N	-	4.75	146.1	87.1	+	38	21
Acetylsalicylic acid	$C_9H_8O_4$	180.15742	C01405	HMDB01879	BSYNRYMUTXBXSQ-UHFFFAOYSA-N	11126-35-5	30.1	180.96	120.96	+	16	13
Adenine	$C_5H_5N_5$	135.1267	C00147	HMDB00034	GFFGJBXGBJISGV-UHFFFAOYSA-N	70700-30-0	6.71	135.88	119	+	25	33
Adenosine	$C_{10}H_{13}N_5O_4$	267.24132	C00212	HMDB00050	OIRDTQYFTABQOQ-KQYNXXCUSA-N	46946-45-6	16.97	268.24	136	+	56	25
Adenosine-5-triphosphate	$C_{10}H_{16}N_5O_{13}P_3$	551.144685	C00002	HMDB00538	TTWYZDPBDWHJOR-UHFFFAOYSA-L	16488-07-6	17.35	506.1	158.8	-	-100	-47
Adenosine-3-monophosphate	$C_{10}H_{14}N_5O_7P$	347.221222	C01367	HMDB03540	LNQVTSROQXJCDD-KQYNXXCUSA-N	-	17.33	348.22	136	+	43	33
Adonitol	$C_5H_{12}O_5$	152.14578	C00474	HMDB00508	HEBKCHPVOIAQTA-ZXFHETKHSA-N	-	27.07	151	107	-	-42	-30
Agmatine	$C_5H_{14}N_4$	130.19146	C00179	HMDB01432	QYPPJABKJHAVHS-UHFFFAOYSA-N	-	57.83	131.1	42.1	+	41	23
Alanine	$C_3H_7NO_2$	89.09318	C01401	-	QNAYBMKLOCPYGJ-UHFFFAOYSA-N	56-41-7	4.58	90	44	+	35	21
Allantoin	$C_4H_6N_4O_3$	158.11544	C01551	HMDB00462	POJWUDADGALRAB-UHFFFAOYSA-N	-	5.78	159.1	116.2	+	26	15
Alpha-D-Mannose-1-phosphate	$C_6H_{13}O_9P$	260.135782	C00636	HMDB06330	HXXFSFRBOHSIMQ-RWOPYEJCSA-N	-	7.53	259	96.9	-	-45	-25
Alpha-lactose	$C_{12}H_{22}O_{11}$	342.29648	C00243	-	GUBGYTABKSRVRQ-XLOQQCSPSA-N	-	34.7	303.1	153.2	+	42	39
alpha-Lipoamide	$C_8H_{15}NOS_2$	205.34	C00248	HMDB00962	FCCDDURTIIUXBY-UHFFFAOYSA-N	940-69-2	20.53	206.1	189.1	+	34	31
Alpha-methyl-DL-serine	$C_4H_9NO_3$	119.11916	C02115	-	CDUUKBXTEOFITR-UHFFFAOYSA-N	-	4.2	120.058	102	+	39	9
Anthranilic acid	$C_7H_7NO_2$	137.13598	C00108	HMDB01123	RWZYAGGXGHYGMB-UHFFFAOYSA-N	118-92-3	14.3	138.4	120	+	42	32
Apigenin	$C_{15}H_{10}O_5$	270.2369	C01477	HMDB02124	KZNIFHPLKGYRTM-UHFFFAOYSA-N	520-36-5	34.03	269.1	116.9	-	-86	-50
Apigenin-7-glucoside	$C_{21}H_{20}O_{10}$	432.38	C04608	-	KMOUJOKENFFTPU-QNDFHXLGSA-N	-	27.53	431.1	268	-	-66	-48
Arginine	$C_6H_{14}N_4O_2$	174.20096	C00062	HMDB00517	ODKSFYDXXFIFQN-BYPYZUCNSA-N	74-79-3	4.75	175	70	+	30	33
Ascorbate	$C_6H_8O_6$	176.12412	C00072	HMDB00044	CIWBSHSKHKDKBQ-JLAZNSOCSA-N	57606-40-3	10.2	177	141	+	28	13
Asparigine	$C_4H_8N_2O_3$	132.11792	C00152	HMDB00168	DCXYFEDJOCDNAF-REOHCLBHSA-N	-	4.58	133	74	+	40	25
Aspartic acid	$C_4H_7NO_4$	133.10268	C00402	HMDB06483	CKLJMWTZIZZHCS-UWTATZPHSA-N	1783-96-6	4.79	134	74	+	35	20
Benzoic acid	$C_7H_6O_2$	122.12134	C00180	HMDB01870	WPYMKLBDIGXBTP-UHFFFAOYSA-N	-	59.2	123.1	79.1	+	32	17
Betaine	$C_5H_{11}NO_2$	117.14634	C00719	HMDB00043	KWIUHFFTVRNATP-UHFFFAOYSA-N	-	5.23	118	59	+	56	11
Betaine aldehyde	$C_5H_{12}NO^+$	102.15488	C00576	HMDB01252	SXKNCCSPZDCRFD-UHFFFAOYSA-N	-	3.97	102.1	59.1	+	94	35
Cadaverine	$C_5H_{14}N_2$	102.17806	C01672	HMDB02322	VHRGRCVQAFMJIZ-UHFFFAOYSA-N	-	8.4	103	86.1	+	33	10
Caffeic acid	$C_9H_8O_4$	180.15742	C01481	HMDB01964	QAIPRVGONGVQAS-DUXPYHPUSA-N	331-39-5	25.3	179	135	-	-43	-10
Caffeine	$C_8H_{10}N_4O_2$	194.1906	C07481	HMDB01847	RYYVLZVUVIJVGH-UHFFFAOYSA-N	58-08-2	23.3	195	138	+	55	10
Capsaicin	$C_{18}H_{27}NO_3$	305.41188	C06866	HMDB02227	YKPUWZUDDOIDPM-SOFGYWHQSA-N	912457-62-6	39.9	306	137.1	+	53	30
Carnitine	$C_7H_{15}NO_3$	161.1989	C00487	-	PHIQHXFUZVPYII-UHFFFAOYSA-O	-	13.8	162.1	58.1	+	49	60
Catechin	$C_{15}H_{14}O_6$	290.26806	C06562	HMDB02780	PFTAWBLQPZVEMU-DZGCQCFKSA-N	-	24.5	291.1	139.1	+	45	27
Cellobiose	$C_{12}H_{22}O_{11}$	342.29648	C00185	HMDB00055	GUBGYTABKSRVRQ-CUHNMECISA-N	-	5.63	341.2	101	-	-40	-34
Chlorogenic Acid	$C_{16}H_{18}O_9$	354.30872	C00852	HMDB03164	CWVRJTMFETXNAD-JUHZACGLSA-N	-	23.6	353	191	-	-51	-20
Chlorophyll	$C_{55}H_{72}MgN_4O_5$	893.48898	C05306	-	ATNHDLDRLWWWCB-AENOIHSZSA-M	-	27.6	447.2	300	-	-78	-43
Choline	$C_5H_{14}NO^+$	104.17076	C00114	HMDB00097	OEYIOHPDSNJKLS-UHFFFAOYSA-N	-	18.57	113	69.1	-	-33	-16
cis,cis-Muconic acid	$C_6H_6O_4$	142.10944	C02480	HMDB06331	TXXHDPDFNKHHGW-CCAGOZQPSA-N	-	5.35	141.11	97	-	-40	-9
cis-4-Hydroxy-L-proline	$C_5H_9NO_3$	131.13	C01015	HMDB06055	PMMYEEVYMWASQN-IMJSIDKUSA-N	618-27-9	4.78	132.2	85.9	+	51	21
cis-Aconitic acid	$C_6H_6O_6$	174.10824	C00417	HMDB00072	GTZCVFVGUGFEME-IWQZZHSRSA-N	-	4.54	175.11	157	+	34	13
Citraconic acid	$C_5H_6O_4$	130.09874	C02226	HMDB00634	HNEGQIOMVPPMNR-IHWYPQMZSA-N	-	21.83	131.04	113.04	+	15	13
Citramalic acid	$C_5H_8O_5$	148.11402	C00815	HMDB00426	XFTRTWQBIOMVPK-UHFFFAOYSA-N	-	16.57	146.8	86.9	-	-37	-21
Citric acid	$C_6H_8O_7$	192.12352	C00158	HMDB00094	KRKNYBCHXYNGOX-UHFFFAOYSA-N	-	59.8	193.32	175.1	+	22	9
Coniferyl aldehyde	$C_{10}H_{10}O_3$	178.1846	C02666	-	DKZBBWMURDFHNE-NSCUHMNNSA-N	-	33.7	179.2	146.8	-	-40	-40
Coumaric acid	$C_9H_8O_3$	164.15802	C12621	HMDB01713	NGSWKAQJJWESNS-ZZXKWVIFSA-N	588-30-7	29.83	165.1	147	+	35	16
Creatine	$C_4H_9N_3O_2$	131.13316	C00300	HMDB00064	CVSVTCORWBXHQV-UHFFFAOYSA-N	-	4.7	132.2	89.9	+	27	35
Creatinine	$C_4H_7N_3O$	113.11788	C00791	HMDB00562	DDRJAANPRJIHGJ-UHFFFAOYSA-N	-	52.43	114	43.8	+	56	30
Curcumin	$[HOC_6H_3(OCH_3)CH=($	368.38	C10443	HMDB02269	ZIUSSTSXXLLKKK-KOBPDPAPSA-N	-	39.47	367.3	133.9	-	-64	-49
Cyanidin chloride	$C_{15}H_{11}ClO_6$	322.69724	C05905	HMDB02708	VEVZSMAEJFVWIL-UHFFFAOYSA-O	-	26.1	287.1	137	+	123	48
Cyclic adenosine diphosphate-ribose	$C_{15}H_{21}N_5O_{13}P_2$	541.3	C13050	-	BQOHYSXSASDCEA-KEOHHSTQSA-L	119340-53-3	17.8	542.04	524.2	+	53	22
Cystathione	$C_7H_{14}N_2O_4S$	222.26206	C02291	HMDB00099	ILRYLPWNYFXEMH-WHFBIAKZSA-N	-	4.54	223	134	+	40	10

Cysteamine	C_2H_7NS	77.14868	C01678	HMDB02991	UFULAYFCSOUIOV-UHFFFAOYSA-N	60-23-1	14.5	77.3	45	+	32	23
Cysteine	$C_3H_7NO_2S$	121.15818	C00736	-	XUJNEKJLAYXESH-UHFFFAOYSA-N	-	58.03	122	104	+	40	15
Cytidine	$C_9H_{13}N_3O_5$	243.21662	C00475	HMDB00089	UHDGCWIWMRVCDJ-XVFCMESISA-N	-	6.04	244.085	112	+	37	17
Cytosine	$C_4H_5N_3O$	111.102	C00380	HMDB00630	OPTASPLRGRRNAP-UHFFFAOYSA-N	71-30-7	4.8	112.043	43	+	61	33
Daidzein	$C_{15}H_{10}O_4$	254.2375	C10208	HMDB03312	ZQSIJRDFPHDXIC-UHFFFAOYSA-N	486-66-8	34.83	237	208	-	-92	-45
Daidzein-7-O-β-D-glucopyranoside	$C_{21}H_{20}O_9$	416.3781	C10216	-	XIVFQYWMMJWUCD-VSTJRZLJSA-N	-	30.9	400.9	189	-	-104	-41
D-alanyl-D-alanine	$C_6H_{12}N_2O_3$	160.17108	C00993	HMDB03459	DEFJQIDDEAULHB-SYPWQXSBSA-N	923-16-0	4.47	161.3	90	+	38	15
Dehydroascorbic acid	$C_6H_6O_6$	174.10824	C00425	HMDB01264	SBJKKFFYIZUCET-DOAHDZERSA-N	-	4.73	175.1	88	+	34	28
D-Glucosamine-6-phosphate	$C_6H_{14}NO_8P$	259.151022	C00352	HMDB01254	AEJSSXDYDSUOOZ-SLPGGIOYSA-N	-	4.59	260	126	+	38	23
D-Glucose-6-phosphate	$C_6H_{13}Na_2O_{10}P$	322.114721	C00352	HMDB01254	UUWJZXLTPORJKW-UHFFFAOYSA-L	-	7.54	259	96.8	-	-44	-74
D-glucuronic acid	$C_6H_9NaO_7$	216.121229	C00191	HMDB00127	WNFHGZLVUQBPMA-JSCKKFHOSA-M	-	5.71	193.1	113.1	-	-29	-28
Diethanolamine	$C_4H_{11}NO_2$	105.13564	C06772	HMDB04437	ZBCBWPMODOFKDW-UHFFFAOYSA-N	-	4.22	106	88	+	38	14
Dihydrofolic acid	$C_{19}H_{21}N_7O_6$	443.41334	C00415	HMDB01056	OZRNSSUDZOLUSN-LBPRGKRZSA-N	-	22.03	440.1	174.8	-	-76	-54
Dihydrophaseic acid	$C_{15}H_{22}O5$	282.33218	C15971	-	KJQFBVYMGADDTQ-UHFFFAOYSA-N	-	41	285.1	267.2	+	32	13
DL-2-Aminoadipic acid	$C_6H_{11}NO_4$	161.15584	C00956	HMDB00510	OYIFNHCXNCRBQI-BYPYZUCNSA-N	-	21.73	162.1	98.1	+	41	25
DL-3-Aminoisobutyric acid	$C_4H_9NO_2$	103.11976	C03284	HMDB02166	QCHPKSFMDHPSNR-VKHMYHEASA-N	-	4.46	104.12	86	+	65	8
DL-Buthionine-sulfoximine	$C_8H_{18}N_2O_3S$	222.31	C04543	-	KJQFBVYMGADDTQ-UHFFFAOYSA-N	-	58.1	229.96	122.08	+	32	22
DL-Homocysteine	$C_4H_9NO_2S$	135.18476	C05330	HMDB00742	FFFHZYDWPBMWHY-UHFFFAOYSA-N	-	5.05	136.18	118	+	41	11
D-Mannitol	$C_6H_{14}O_6$	182.17176	C00392	HMDB00765	FBPFZTCFMRRESA-KVTDHHQDSA-N	69-65-8	5.02	183.1	147.1	+	24	13
D-mannose-6-phosphate	$C_6H_{11}O_9P^{-2}$	258.119902	C00275	HMDB01078	NBSCHQHZLSJFNQ-QTVWNMPRSA-N	-	7.53	259	96.9	-	-49	-40
Dodecanoic acid	$C_{12}H_{24}O_2$	200.31776	C02679	HMDB00638	POULHZVOKOAJMA-UHFFFAOYSA-N	-	7.55	199.1	79	-	-74	-61
D-Ribose-5-phosphate	$C_5H_{11}O_8P$	230.109802	C00117	HMDB01548	KTVPXOYAKDPRHY-SOOFDHNKSA-N	-	22.27	228.9	96.9	-	-30	-34
Epicatechin	$C_{15}H_{14}O_6$	290.26806	C09727	HMDB01871	PFTAWBLQPZVEMU-UKRRQHHQSA-N	490-46-0	24.5	291.1	139.1	+	54	26
Eriodictyol	$C_{15}H_{12}O_6$	288.25218	C05631	HMDB05810	SBHXYTNGIZCORC-ZDUSSCGKSA-N	-	23.2	289.25	243	+	30	14
Ethanolamine	C_2H_7NO	61.08308	C00189	HMDB00149	HZAXFHJVJLSVMW-UHFFFAOYSA-N	20989-17-7	4.54	62	44	+	30	8
Ethylmalonic acid	$C_5H_8O_4$	132.11462	C02170	HMDB00202	UKFXDFUAPNAMPJ-UHFFFAOYSA-N	-	22.27	131.1	87.1	-	-52	-34
Ferulic acid	$C_{10}H_{10}O$	194.184	C01494	-	KSEBMYQBYZTDHS-HWKANZROSA-N	537-98-4	28.3	195.12	177	+	32	17
Folic acid	$C_{19}H_{19}N_7O_6$	441.39746	C00504	HMDB00121	OVBPIULPVIDEAO-LBPRGKRZSA-N	59-30-3	21.9	442	295	+	41	28
Fumaric acid	$C_4H_4O_4$	116.07216	C00122	HMDB00134	VZCYOOQTPOCHFL-OWOJBTEDSA-N	623158-97-4	20.9	115.1	70.9	-	-29	-22
Galactinol	$C_{12}H_{22}O_{11}$	342.29648	C01235	HMDB11739	VCWMRQDBPZKXKG-ZNVDUFQESA-N	-	37.43	341.1	79.2	-	-20	-63
Gallic acid	$C_7H_6O_5$	170.11954	C01424	HMDB05807	LNTHITQWFMADLM-UHFFFAOYSA-N	-	4.88	171.12	127	+	24	16
gamma-L-glutamyl-L-cysteine	$C_8H_{14}N_2O_5S$	250.27216	C00669	HMDB01049	RITKHVBHSGLULN-WHFBIAKZSA-N	-	4.83	147	84	+	40	19
Genistein	$C_{15}H_{10}O_5$	270.2369	C09126	-	BKAWJIRCKVUVED-UHFFFAOYSA-N	-	33.97	271.1	91	+	36	49
Gentisic acid	$C_7H_6O_4$	154.12014	C00628	HMDB00152	WXTMDXOMEHJXQO-UHFFFAOYSA-N	-	1.37	177.12	159	+	22	13
Gibberellin A3	$C_{19}H_{22}O_6$	346.37438	C01699	HMDB03559	IXORZMNAPKEEDV-OBDJNFEBSA-N	-	26.5	347.28	311.2	+	24	24
Gibberellin A4	$C_{20}H_{25}O_5^-$	345.4095	C11864	-	KSBJAONOPKRVRR-YTJHIPEWSA-M	-	35.65	333.12	315.12	+	22	15
Glucose	$C_6H_{12}O_6$	180.15588	C00031	HMDB00122	WQZGKKKJIJFFOK-DVKNGEFBSA-N	-	25.6	181.2	163.1	+	14	9
Glutamic acid	$C_5H_9NO_4$	147.12926	C00064	HMDB00641	WHUUTDBJXJRKMK-UHFFFAOYSA-N	-	4.84	148	84	+	35	25
Glutamine	$C_5H_{10}N_2O_3$	146.1445	C00064	HMDB00641	ZDXPYRJPNDTMRX-VKHMYHEASA-N	-	3.67	147	130	+	36	9
Glutaric acid	$C_5H_8O_4$	132.11462	C00489	HMDB00661	JFCQEDHGNNZCLN-UHFFFAOYSA-N	-	4.94	133.1	87	+	47	18
GSSG	$C_{20}H_{32}N_6O_{12}S_2$	612.63108	C00127	HMDB03337	YPZRWBKMTBYPTK-BJDJZHNGSA-N	-	16.8	613.2	355.1	+	47	35
GSH	$C_{10}H_{17}N_3O_6S$	307.32348	C00051	HMDB00125	RWSXRVCMGQZWBV-WDSKDSINSA-N	70-18-8	16.9	308.4	179	+	22	22
Glycine	$C_2H_5NO_2$	75.0666	C00037	HMDB00123	DHMQDGOQFOQNFH-UHFFFAOYSA-N	848646-45-7	58.1	76	30	+	30	20
Glycolic acid	$C_2H_4O_3$	76.05136	C00160	HMDB00115	AEMRFAOFKBGASW-UHFFFAOYSA-N	79-14-1	6.57	75	47.1	-	-27	-16
Guanidine	CH_6ClN_3	95.53144	C00179	HMDB01842	PJJJBBJSCAKJQF-UHFFFAOYSA-N	-	59.3	97.08	69	+	53	69
Guanine	$C_5H_5N_5O$	151.1261	C00242	HMDB00132	UYTPUPDQBNUYGX-UHFFFAOYSA-N	73-40-5	7.8	151.6	135	+	85	30
Guanosine	$C_{10}H_{13}N_5O_5$	283.24072	C00387	HMDB00133	NYHBQMYGNKIUIF-UUOKFMHZSA-N	118-00-3	17.8	284	152	+	35	14
Guanosine-3,5-cyclicphosphate	$C_{10}H_{12}N_5O_7P$	345.205342	C00942	HMDB01314	ZOOGRGPOEVQQDX-UUOKFMHZSA-N	-	21.4	346.08	152.04	+	39	27
Gulonic acid gamma lactone	$C_6H_{10}O_6$	178.14	C01040	HMDB03466	SXZYCXMUPBBULW-SKNVOMKLSA-N	-	52.3	176.7	59	-	-46	-31
Hemineurine	C_6H_9NOS	143.20676	C04294	-	BKAWJIRCKVUVED-UHFFFAOYSA-N	-	18.2	146.3	114	+	47	50
Hesperetin	$C_{16}H_{14}O_6$	302.27876	C01709	HMDB05782	AIONOLUJZLIMTK-AWEZNQCLSA-N	520-33-2	34.43	301.3	135.9	-	-84	-48
Histamine	$C_5H_9N_3$	111.14506	C00388	HMDB00870	NTYJJOPFIAHURM-UHFFFAOYSA-N	51-45-6	4.46	112	95	+	38	22
Histidine	$C_6H_9N_3O_2$	155.15456	C00135	HMDB00177	HNDVDQJCIGZPNO-YFKPBYRVSA-N	-	30	156	110	+	40	14
Homogentisic acid	$C_8H_8O_4$	168.14672	C00544	HMDB00130	IGMNYECMUMZDDF-UHFFFAOYSA-N	-	19.7	167	121.9	-	-31	-33
Hypotaurine	$C_2H_7NO_2S$	109.14748	C00519	HMDB00965	VVIUBCNYACGLLV-UHFFFAOYSA-N	-	28.5	110.1	63	+	60	30
Hypoxanthine	$C_5H_4N_4O$	136.11146	C00262	HMDB00157	FDGQSTZJBFJUBT-UHFFFAOYSA-N	68-94-0	14.65	135	92	-	-64	-25
Indole-3-butyric acid	$C_{12}H_{13}NO_2$	203.23712	C11284	HMDB02096	JTEDVYBZBROSJT-UHFFFAOYSA-N	133-32-4	34.1	204.12	186.1	+	26	20
Indole carboxylic acid	$C_9H_7NO_4$	193.15618	C04185	HMDB01253	YFTGOBNOJKXZJC-UHFFFAOYSA-N	1055767	29.37	162	118	+	48	20
Indole-3-acetic acid	$C_{10}H_9NO_2$	175.18396	C00954	HMDB00197	SEOVTRFCIGRIMH-UHFFFAOYSA-N	87-51-4	31	176	129.9	+	53	26
Inosine	$C_{10}H_{12}N_4O$	268.22608	C00294	HMDB00195	UGQMRVRMYYASKQ-KQYNXXCUSA-N	-	17.9	269.23	137	+	36	21
Inosine-5-diphosphate	$C_{10}H_{14}N_4O_{11}P_2$	428.185884	C00104	HMDB03335	JPXZQMKKFWMMGK-KQYNXXCUSA-N	-	23.3	427	134.9	-	-61	-36
Isomaltose	$C_{12}H_{22}O_{11}$	342.29648	C00252	HMDB02923	DLRVVLDZNNYCBX-RTPHMHGBSA-N	-	57.97	341.1	182.8	-	-29	-56

Itaconic acid	$C_5H_6O_4$	130.09874	C00490	HMDB02092	LVHBHZANLOWSRM-UHFFFAOYSA-N	-	22.6	129	84.9	-	-29	-8
Jasmonic acid	$C_{12}H_{18}O_3$	210.26952	C08491	-	ZNJFBWYDHIGLCU-HWKXXFMVSA-N	-	33.9	211.27	193	+	39	12
Kaempferide	$C_{16}H_{12}O_6$	300.26	C10098	-	SQFSKOYWJBQGKQ-UHFFFAOYSA-N	491-54-3	26	301.3	153	+	94	51
Kaempferol	$C_{15}H_{10}O_6$	286.2363	C05903	HMDB05801	IYRMWMYZSQPJKC-UHFFFAOYSA-N	-	31.1	285.2	117	-	-29	-50
Kinetin	$C_{10}H_9N_5O$	215.21136	C08272	HMDB12245	QANMHLXAZMSUEX-UHFFFAOYSA-N	525-79-1	21.87	216.3	81.1	+	49	32
L-Dihydroorotic acid	$C_5H_6N_2O_4$	158.11214	C00337	HMDB03349	UFIVEPVSAGBUSI-REOHCLBHSA-N	-	23.7	157.1	112.9	-	-87	-16
L-Glutathione	$C_{10}H_{17}N_3O_6S$	307.32348	C14863	-	RWSXRVCMGQZWBV-WDSKDSINSA-N	-	16.77	307.1	185	+	73	44
L-Homocysteine	$C_4H_9NO_2S$	135.18476	C05330	HMDB00742	FFFHZYDWPBMWHY-UHFFFAOYSA-N	-	5.2	136.2	56.1	+	98	32
L-2-Amino-3-guanidinopropionic acid	$C_4H_{11}ClN_4O_2$	182.60874	C03065	-	ZOXYHKHLLCDEAX-DKWTVANSSA-N	-	4.81	132	72.1	+	40	24
L-beta-Homomethionine	$C_6H_{13}NO_2S$	163.23792	C17213	-	QWVNCDVONVDGDV-UHFFFAOYSA-N	-	10.6	164.2	104	+	35	29
L-Carnosine	$C_9H_{14}N_4O_3$	226.23246	C00386	HMDB00033	CQOVPNPJLQNMDC-ZETCQYMHSA-N	305-84-0	18.2	227.1	110.1	+	49	39
L-Citrulline	$C_6H_{13}N_3O_3$	175.18572	C00327	HMDB00904	RHGKLRLOHDJJDR-BYPYZUCNSA-N	372-75-8	4.88	176.1	70	+	35	35
L-Cystathionine	$C_7H_{14}N_2O_4S$	222.26206	C02291	HMDB00099	ILRYLPWNYFXEMH-WHFBIAKZSA-N	-	4.53	223.1	134.1	+	39	44
L-Cysteine-S-sulfate	$C_3H_7NO_5S_2$	201.22138	C05824	HMDB00731	NOKPBJYHPHHWAN-REOHCLBHSA-N	-	7.69	199.8	136	-	-42	-27
Leucine/Isoleucine	$C_6H_{13}NO_2$	131.17292	C00407	HMDB00172	AGPKZVBTJJNPAG-WHFBIAKZSA-N	443-79-8	5	132	86	+	40	9
L-Malic acid	$C_4H_6O_5$	134.08744	C00149	HMDB00156	BJEPYKJPYRNKOW-REOHCLBHSA-N	-	4.78	135	89	+	19	10
L-Methionine sulfone	$C_5H_{11}NO_4S$	181.21014	_	HMDB02005	UCUNFLYVYCGDHP-BYPYZUCNSA-N	7314-32-1	5.22	182	56	+	38	30
L-Ornithine	$C_5H_{12}N_2O_2$	132.16098	C00077	HMDB00214	AHLPHDHHMVZTML-BYPYZUCNSA-N	-	4.57	133	69.9	+	40	28
Lutein	$C_{40}H_{56}O_2$	568.87144	C08601	HMDB03233	KBPHJBAIARWVSC-RGZFRNHPSA-N	-	44.7	351.2	271.1	-	-33	-11
Luteolin	$C_{15}H_{10}O_6$	286.2363	C01514	HMDB05800	IQPNAANSBPBGFQ-UHFFFAOYSA-N	-	31.87	285.2	133	-	-73	-48
Luteolin-7-beta-D-glucoside	$C_{21}H_{20}O_{11}$	448.38	C03951	-	OXGUCUVFOIWWQJ-HQBVPOQASA-N	-	26.1	449.38	287	+	51	21
Malate	$C_4H_6O_5$	134.08744	C00497	-	BJEPYKJPYRNKOW-UWTATZPHSA-N	636-61-3	5.03	135.1	117	+	15	15
Malonic acid	$C_3H_4O_4$	104.06146	C00383	HMDB00691	OFOBLEOULBTSOW-UHFFFAOYSA-N	-	10.08	103	59.2	-	-23	-15
Malonyl CoA	$C_{24}H_{38}N_7O_{19}P_3S$	853.580306	C00083	HMDB01175	LTYOQGRJFJAKNA-DVVLENMVSA-N	-	0.58	734.3	692.3	-	-68	-50
Maltose	$C_{12}H_{22}O_{11}$	342.29648	C00208	HMDB00163	GUBGYTABKSRVRQ-PICCSMPSSA-N	-	4.45	341	59.1	-	-27	-44
Mandelic acid	$C_8H_8O_3$	152.14732	C01984	HMDB00703	IWYDHOAUDWTVEP-ZETCQYMHSA-N	17199-29-0	27.07	151.1	107	-	-64	-14
Melatonin	$C_{13}H_{16}N_2O_2$	232.27834	C01598	HMDB01389	DRLFMBDRBRZALE-UHFFFAOYSA-N	73-31-4	29.27	233	174.1	+	42	35
Melibiose	$C_{12}H_{22}O_{11}$	342.29648	C05402	HMDB00048	DLRVVLDZNNYCBX-ABXHMFFYSA-N	-	7.58	257.1	179	+	23	17
Mesaconic acid	$C_5H_6O_4$	130.09874	C01732	HMDB00749	HNEGQIOMVPPMNR-NSCUHMNNSA-N	-	22.23	128.9	84.8	-	-12	-20
Metheonine	$C_5H_{11}NO_2S$	149.21134	C00073	HMDB00696	FFEARJCKVFRZRR-BYPYZUCNSA-N	2899-37-8	58.13	150	104	+	40	20
Methylmalonic acid	$C_4H_6O_4$	118.08804	C02170	HMDB00202	ZIYVHBGGAOATLY-UHFFFAOYSA-N	-	17.97	116.9	55	-	-34	-41
Methylthiobutyric acid	$C_5H_8O_3S$	148.18022	C01180	HMDB01553	SXFSQZDSUWACKX-UHFFFAOYSA-N	-	28.83	171.12	153.1	+	23	13
Myricetin	$C_{15}H_{10}O_8$	318.2351	C10107	HMDB02755	IKMDFBPHZNJCSN-UHFFFAOYSA-N	-	46.1	317.23	151	-	-65	-10
N,N-dimethylglycine	$C_4H_9NO_2$	103.11976	C01026	HMDB00092	FFDGPVCHZBVARC-UHFFFAOYSA-N	-	4.85	104	58.1	+	43	24
N-acetyl-D-mannosamine	$C_8H_{15}NO_6$	221.2078	C00645	HMDB11744	OVRNDRQMDRJTHS-ZTVVOAFPSA-N	-	17.5	222.2	126.1	+	34	29
N-Acetylneuraminic acid	$C_{11}H_{19}NO_9$	309.26986	C00270	HMDB00230	SQVRNKJHWKZAKO-LUWBGTNYSA-N	-	7.3	310.1	273.9	+	37	36
N-Alpha-L-ornithine	$C_7H_{14}N_2O_3$	174.19766	C00437	HMDB03357	JRLGPAXAGHMNOL-LURJTMIESA-N	-	4.57	133	70.1	+	74	26
Naringenin	$C_{15}H_{12}O_5$	272.25278	C00509	HMDB02670	FTVWIRXFELQLPI-ZDUSSCGKSA-N	-	33.9	271.1	150.8	-	-60	-29
Naringin	$C_{27}H_{32}O_{14}$	580.53458	C00509	HMDB02670	DFPMSGMNTNDNHN-JJLSSNRUSA-N	-	26.83	579.53	271.5	-	-29	-50
Neohesperidin	$C_{28}H_{34}O_{15}$	610.56056	C12630	HMDB05799	ARGKVCXINMKCAZ-UZRWAPQLSA-N	-	27.23	609.4	301.1	-	-16	-54
N-formyl-L-methionine	$C_6H_{11}NO_3S$	177.22144	C03145	HMDB01015	PYUSHNKNPOHWEZ-YFKPBYRVSA-N	-	23	178.1	103.9	+	36	24
Niacinamide	$C_6H_6N_2O$	122.12464	C00153	HMDB01406	DFPAKSUCGFBDDF-UHFFFAOYSA-N	63748-44-7	10.8	123	80	+	55	11
Nicotinamide	$C_6H_6N_2O$	122.12464	C00153	HMDB01406	DFPAKSUCGFBDDF-UHFFFAOYSA-N	63748-44-7	10.7	123.1	79.8	+	59	32
Nicotine	$C_{10}H_{14}N_2$	162.23156	C00745	HMDB01934	SNICXCGAKADSCV-JTQLQIEISA-N	54-11-5	59.4	163.23	117	+	31	12
Nicotinic acid	$C_6H_5NO_2$	123.1094	C00253	HMDB01488	PVNIIMVLHYAWGP-UHFFFAOYSA-N	59-67-6	8.87	124	96	+	60	17
Norepinephrine	$C_8H_{11}NO_3$	169.17784	C00547	HMDB00216	SFLSHLFXELFNJZ-QMMMGPOBSA-N	4899-05_2	7.7	170	107.1	+	29	53
O-Acetyl-L-serine	$C_5H_9NO_4$	147.12926	C00979	HMDB03011	VZXPDPZARILFQX-BYPYZUCNSA-N	-	4.92	148	88	+	29	28
O-hydroxycinnamic acid	$C_9H_8O_3$	164.15802	C03549	HMDB02641	PMOWTIHVNWZYFI-AATRIKPKSA-N	-	29.83	165.12	147	+	25	16
O-phospho Serine	$C_3H_8NO_6P$	185.072482	C01005	HMDB00272	BZQFBWGGLXLEPQ-REOHCLBHSA-N	-	6.47	184	78.8	-	-31	-34
Orotic Acid	$C_5H_4N_2O_4$	156.09626	C00295	HMDB00226	PXQPEWDEAKTCGB-UHFFFAOYSA-N	65-86-1	7.5	157	79	+	47	36
O-Succinyl-L-homoserine	$C_8H_{13}NO_6$	219.19192	C01118	-	GNISQJGXJIDKDJ-YFKPBYRVSA-N	-	8.61	220.1	101.9	+	72	32
Oxalic acid	$C_2H_2O_4$	90.03488	C00209	HMDB02329	MUBZPKHOEPUJKR-UHFFFAOYSA-N	-	19.4	89	42.2	-	-26	-77
Palmitic acid	$C_{16}H_{32}O_2$	256.42408	C00249	HMDB00220	IPCSVZSSVZVIGE-UHFFFAOYSA-N	-	31.43	255.2	96.6	-	-24	-25
Pelargonin chloride	$C_{27}H_{31}ClO_{15}$	630.98	C08725	-	ZKHQWZAMYRWXGA-KQYNXXCUSA-N	-	25.8	593.3	269.3	-	-85	-43
Phenylalanine	$C_9H_{11}NO_2$	165.18914	C00079	HMDB00159	COLNVLDHVKWLRT-QMMMGPOBSA-N	3182-95-4	18.23	166	120	+	28	16
Pimelic acid	$C_7H_{12}O_4$	160.16778	C02656	HMDB00857	WLJVNTCWHIRURA-UHFFFAOYSA-N	111-16-0	24.37	159.1	96.8	-	-54	-29
Proline	$C_5H_9NO_2$	115.13046	C00148	HMDB00162	ONIBWKKTOPOVIA-BYPYZUCNSA-N	18875-45-1	5.3	116	70	+	60	17
Putrescine	$C_4H_{12}N_2$	88.15148	C00134	HMDB01414	KIDHWZJUCRJVML-UHFFFAOYSA-N	-	52.4	89.1	72	+	35	11
Pyridoxal-5-phosphate	$C_8H_9NO_6P^-$	246.133922	C00627	HMDB01319	NGVDGCNFYWLIFO-UHFFFAOYSA-M	-	17.5	248.1	150.1	+	50	28
Pyridoxine	$C_8H_{11}NO_3$	169.17784	C00314	HMDB00239	LXNHXLLTXMVWPM-UHFFFAOYSA-N	8059-24-3	8.61	170.18	152	+	64	8
Pyrocatechol	$C_6H_6O_2$	110.11064	C00090	HMDB00957	YCIMNLLNPGFGHC-UHFFFAOYSA-N	-	28.07	108.9	91	-	-61	-27
pyroglutamic acid	$C_5H_7NO_3$	129.11398	C01879	HMDB00267	ODHCTXKNWHHXJC-VKHMYHEASA-N	98-79-3	14.3	129.8	84	+	40	20

Quercetin	$C_{15}H_{10}O_7$	302.2357	C00389	HMDB05794	REFJWTPEDVJJIY-UHFFFAOYSA-N	117-39-5	28.2	301.24	273	-	-70	-28
Quercetin-3-O-rhamnoside	$C_{21}H_{20}O_{11}$	448.3769	C01750	-	XJLXINKUBYWONI-NNYOXOHSSA-O	-	27.6	447.3	300.1	-	-77	-40
Raffinose	$C_{18}H_{32}O_{16}$	504.43708	C00492	HMDB03213	MUPFEKGTMRGPLJ-ZQSKZDJDSA-N	-	16.5	503.4	179.1	-	-104	-40
Resveratrol	$C_{14}H_{12}O_3$	228.24328	C03582	HMDB03747	LUKBXSAWLPMMSZ-OWOJBTEDSA-N	501-36-0	30.65	227	143	-	-74	-38
Retinol	$C_{20}H_{30}O$	286.4516	C00473	HMDB00305	FPIPGXGPPPQFEQ-OVSJKPMPSA-N	-	18.95	288.1	270.3	+	47	36
Rhamnose	$C_6H_{12}O_5$	164.15648	C00507	HMDB00849	SHZGCJCMOBCMKK-JFNONXLTSA-N	-	52.7	163.1	59	-	-43	-21
Riboflavin	$C_{17}H_{20}N_4O_6$	376.3639	C00255	HMDB00244	AUNGANRZJHBGPY-SCRDCRAPSA-N	83-88-5	23.2	377.4	243	+	63	24
Riboflavin-5-Monophosphate	$C_{17}H_{21}N_4O_9P$	456.343802	C00061	HMDB01520	FVTCRASFADXXNN-MBNYWOFBSA-N	-	29.65	455.1	255	-	-46	-43
Rosmarinic acid	$C_{18}H_{16}O_8$	360.31484	C01850	HMDB03572	DOUMFZQKYFQNTF-WUTVXBCWSA-N	-	52.5	359.3	160.9	-	-62	-25
Rutin	$C_{27}H_{30}O_{16}$	610.5175	C05625	HMDB03249	IKGXIBQEEMLURG-NVPNHPEKSA-N	-	25.5	609	300	-	-10	-54
S-Adenosyl-L-homocysteine	$C_{14}H_{20}N_6O_5S$	384.4108	C00021	HMDB00939	ZJUKTBDSGOFHSH-WFMPWKQPSA-N	-	16.7	385.3	136.1	+	53	59
Salicin	$C_{13}H_{18}O_7$	286.27782	C01451	HMDB03546	NGFMICBWJRZIBI-UJPOAAIJSA-N	-	21.17	285	120.9	-	-25	-46
Salicylic acid	$C_7H_6O_3$	138.12074	C00805	HMDB01895	YGSDEFSMJLZEOE-UHFFFAOYSA-N	8052-31-1	34.55	139.1	121.1	+	35	21
Sarcosine	$C_3H_7NO_2$	89.09318	C00213	HMDB00271	FSYKKLYZXJSNPZ-UHFFFAOYSA-N	107-97-1	4.57	89.9	44.1	+	50	22
Scopoletin	$C_{10}H_8O_4$	192.17	C01752	-	RODXRVNMMDRFIK-UHFFFAOYSA-N	92-61-5	40.7	191	175	-	-35	-9
Serine	$C_3H_7NO_3$	105.09258	C00065	HMDB00187	MTCFGRXMJLQNBG-REOHCLBHSA-N	56-45-1	59.1	106	60	+	35	8
Serotonin	$C_{10}H_{12}N_2O$	176.21508	C00780	HMDB00259	QZAYGJVTTNCVMB-UHFFFAOYSA-N	-	16.63	177.1	115.1	+	27	38
Shikimic acid	$C_7H_{10}O_5$	174.1513	C00493	HMDB03070	JXOHGGNKMLTUBP-HSUXUTPPSA-N	-	7.8	173	93	-	-51	-24
Sinapic acid	$C_{11}H_{12}O_5$	224.20998	C00482	-	PCMORTLOPMLEFB-ONEGZZNKSA-N	-	27.9	223	208	-	-43	-10
Spermidine	$C_7H_{19}N_3$	145.24586	C00315	HMDB01257	ATHGHQPFGPMSJY-UHFFFAOYSA-N	-	57.5	146.1	72.1	+	41	25
Spermine	$NH_2(CH_2)_3NH(CH_2)_4N$	202.34	C00750	HMDB01256	SNICXCGAKADSCV-UHFFFAOYSA-N	-	32.8	203.2	112.1	+	45	32
Sphingosine	$C_{18}H_{37}NO_2$	299.49188	C00319	HMDB00252	WWUZIQQURGPMPG-KRWOKUGFSA-N	-	46.9	300.5	281.9	+	15	16
Sphingosine-1-phosphate	$C_{18}H_{38}NO_5P$	379.471782	C01120	HMDB01383	DUYSYHSSBDVJSM-KRWOKUGFSA-N	-	18.1	380.3	264.2	+	34	29
Succinic acid	$C_4H_6O_4$	118.08804	C00042	HMDB00254	KDYFGRWQOYBRFD-UHFFFAOYSA-N	623158-99-6	17.97	117	73.1	-	-28	-18
Syringic acid	$C_9H_{10}O_5$	198.1727	C05773	-	JMSVCTWVEWCHDZ-UHFFFAOYSA-N	-	25.3	199.9	141.1	+	50	24
Taurine	$C_2H_7NO_3S$	125.14688	C00245	HMDB00251	XOAAWQZATWQOTB-UHFFFAOYSA-N	-	1.53	126	44.2	+	58	16
Thiamine	$C_{12}H_{17}N_4OS^+$	265.35458	C00378	HMDB00235	JZRWCGZRTZMZEH-UHFFFAOYSA-N	57777-32-9	59.3	264.9	122	+	83	24
Thiamine mono phosphate	$C_{12}H_{18}N_4O_4PS^+$	345.334482	C01081	HMDB02666	HZSAJDVWZRBGIF-UHFFFAOYSA-O	-	4.68	346	224.2	+	50	21
Threonine	$C_4H_9NO_3$	119.11916	C00188	HMDB00167	AYFVYJQAPQTCCC-GBXIJSLDSA-N	-	4.69	120	74	+	32	13
Thymidine	$C_{10}H_{14}N_2O_5$	242.22856	C00214	HMDB00273	IQFYYKKMVGJFEH-XLPZGREQSA-N	50-89-5	19.7	243.09	127.1	+	30	13
Thymine	$C_5H_6N_2O_2$	126.11334	C00178	HMDB00262	RWQNBRDOKXIBIV-UHFFFAOYSA-N	65-71-4	19.23	127	109.9	+	62	25
trans-4-Hydroxy-L-proline	$C_5H_9NO_3$	131.12986	C01157	HMDB00725	PMMYEEVYMWASQN-DMTCNVIQSA-N	-	4.79	132	85.9	+	52	24
Trans-cinnamic acid	$C_9H_8O_2$	148.15862	C10438	HMDB00567	WBYWAXJHAXSJNI-VOTSOKGWSA-N	-	33.2	149.04	131.1	+	24	14
Triethanolamine	$(HOCH_2CH_2)_3N$	149.19	C06771	-	HHLJUSLZGFYWKW-UHFFFAOYSA-N	102-71-6	57.5	150	132	+	59	18
Trimethylamine N-oxide	C_3H_9NO	75.10966	C01104	HMDB00925	UYPYRKYUKCHHIB-UHFFFAOYSA-N	-	4.23	76	59.1	+	43	10
Tropinone	$C_8H_{13}NO$	139.19492	C00783	-	QQXLDOJGLXJCSE-UHFFFAOYSA-N	532-24-1	16.53	140.195	122	+	37	19
Tryptamine	$C_{10}H_{12}N_2$	160.21568	C00398	HMDB00303	FBPFZTCFMRRESA-KVTDHHQDSA-N	61-54-1	20.15	161	144	+	34	10
Tryptophan	$C_{11}H_{12}N_2O_2$	204.22518	C00078	HMDB00929	QIVBCDIJIAJPQS-VIFPVBQESA-N	73-22-3	20.35	205	146	+	43	12
Tyramine	$C_8H_{11}NO$	137.17904	C00483	HMDB00306	DZGWFCGJZKJUFP-UHFFFAOYSA-N	-	9.49	138	121	+	30	12
Tyrosine	$C_9H_{11}NO_3$	181.18854	C00082	HMDB00158	OUYCCCASQSFEME-QMMMGPOBSA-N	140-43-2	5	182	136	+	40	20
Uracil	$C_4H_4N_2O_2$	112.08676	C00106	HMDB00300	ISAKRJDGNUQOIC-UHFFFAOYSA-N	66-22-8	10.6	112.92	96	+	45	25
Uridine	$C_9H_{12}N_2O_6$	244.20138	C00299	HMDB00296	DRTQHJPVMGBUCF-XVFCMESISA-N	58-96-8	16.93	245.069	113	+	33	22
Uridine-5-monophosphate	$C_9H_{13}N_2O_9P$	324.181282	C00105	HMDB00288	DJJCXFVJDGTHFX-XVFCMESISA-N	-	17.15	323.1	78.9	-	-87	-42
Valine	$C_5H_{11}NO_2$	117.14634	C00183	HMDB00883	KZSNJWFQEVHDMF-BYPYZUCNSA-N	2026-48-4	56.9	118	72	+	59	9
Vanillic acid	$C_8H_8O_4$	168.14672	C06672	HMDB00484	WKOLLVMJNQIZCI-UHFFFAOYSA-N	-	8.4	167.4	107.9	-	-50	-20
Vanillin	$C_8H_8O_3$	152.14732	C00755	HMDB12308	MWOOGOJBHIARFG-UHFFFAOYSA-N	121-33-5	27.8	153.3	93.1	+	46	36
Vitexin	$C_{21}H_{20}O_{10}$	432.3775	C01460	-	SGEWCQFRYRRZDC-VPRICQMDSA-N	-	27.7	431.2	311	-	-74	-38
Xanthine	$C_5H_4N_4O_2$	152.11086	C00385	HMDB00292	LRFVTYWOQMYALW-UHFFFAOYSA-N	28522-58-9	31.2	151.1	107.9	-	-48	-25
Xylitol	$C_5H_{12}O_5$	152.14578	C00379	HMDB02917	HEBKCHPVOIAQTA-SCDXWVJYSA-N	-	11.5	153.153	135	+	28	10
Zeatin	$C_{10}H_{13}N_5O$	219.24312	C15545	-	UZKQTCBAMSWPJD-UQCOIBPSSA-N	-	18.57	220.08	202.1	+	41	21
Zeatin riboside	$C_{15}H_{21}N_5O_5$	351.36	C16449	-	GOSWTRUMMSCNCW-HNNGNKQASA-N	-	21.3	351.96	220.2	+	22	25

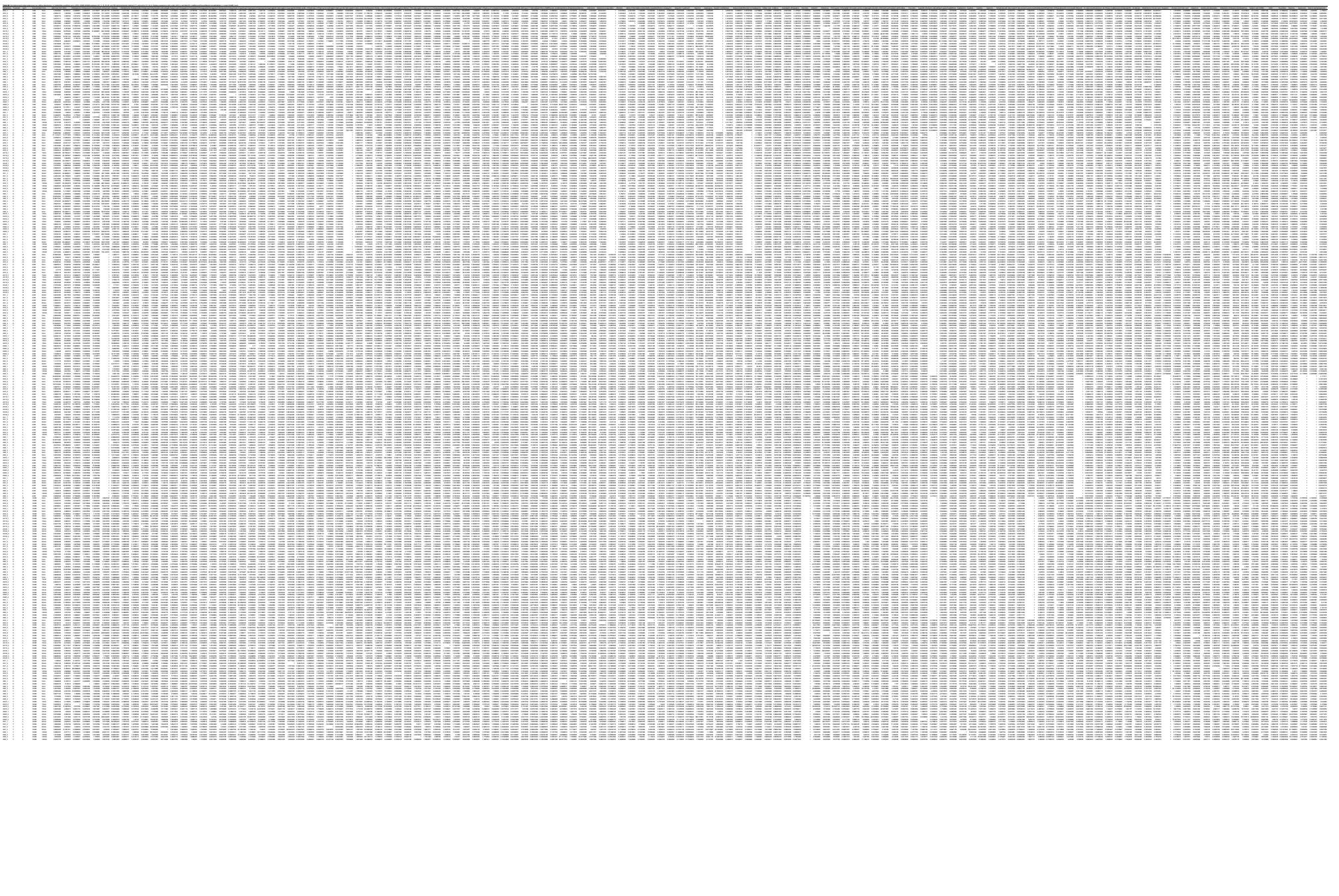

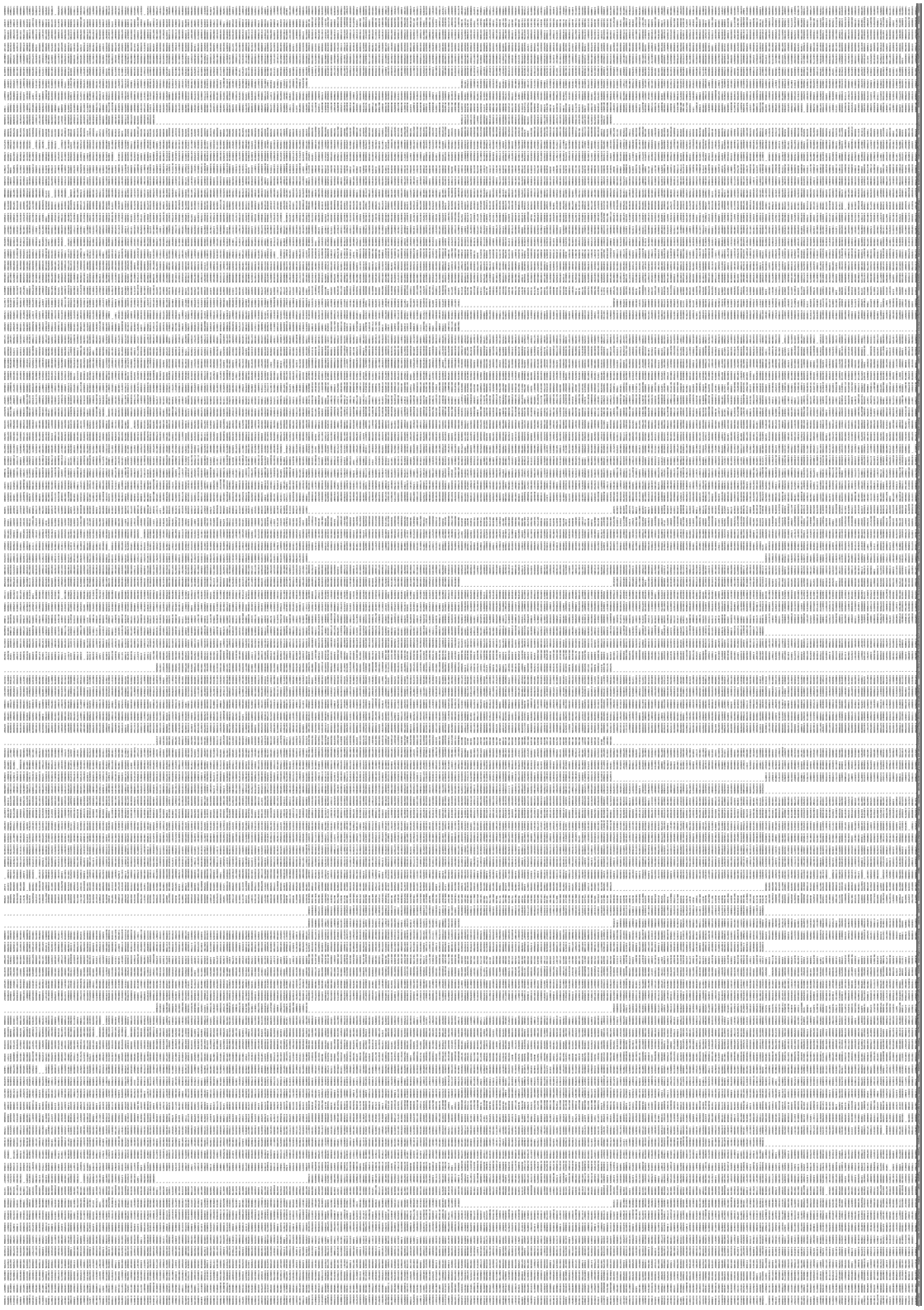

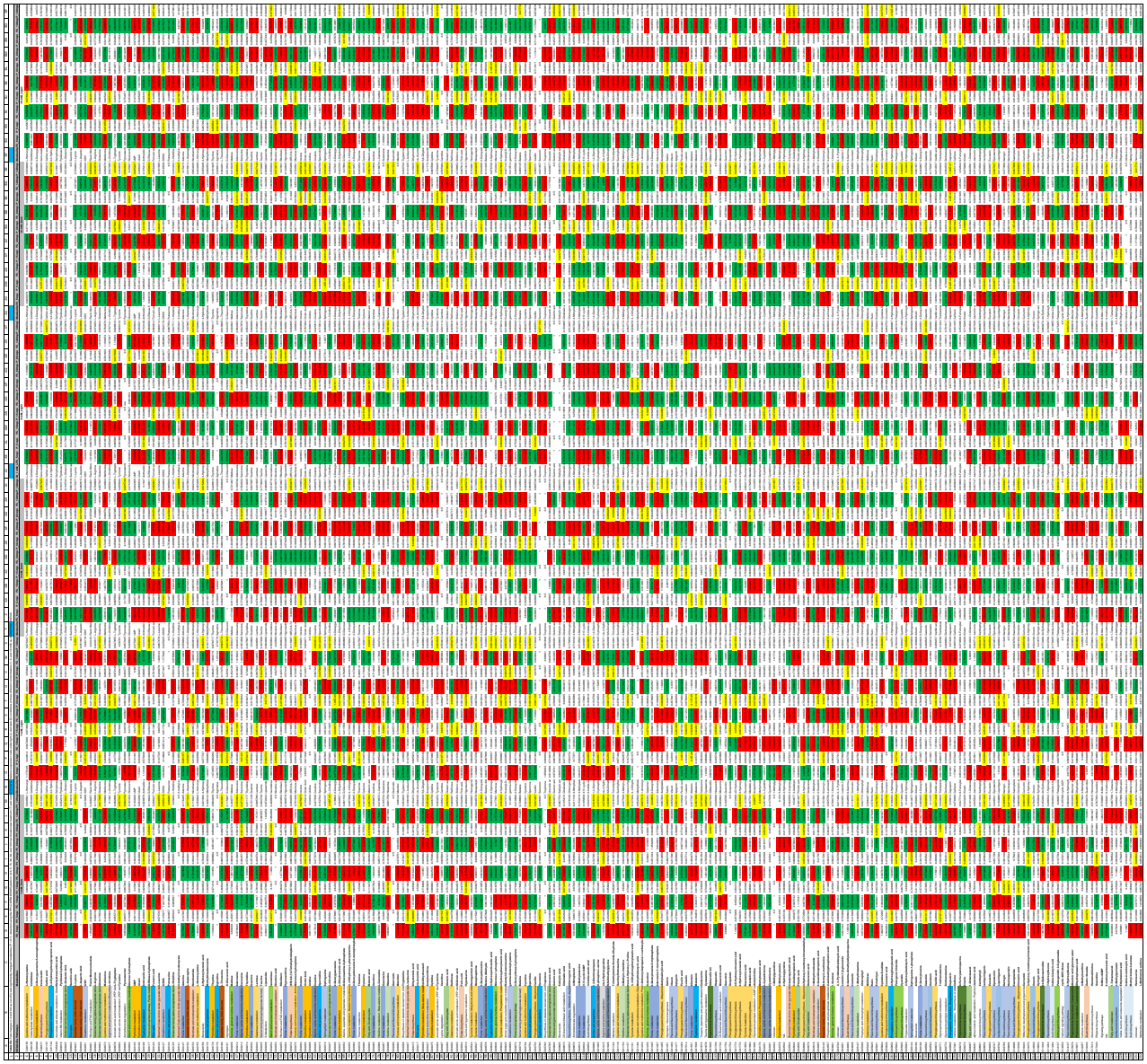

Table S4. Fold changes (1.2 and 0.8 as cut-offs) and significantly changed metabolites (P < 0.1) detected by GC-MS platform at 0, 5, 15, 30, 60, and 120 min bicarbonate treatment (T) and control (C) for A. thaliana suspension cells under light (L) and dark (D) conditions at three different concentrations (1, 3, and 10 mM) as inferred from ANOVA.

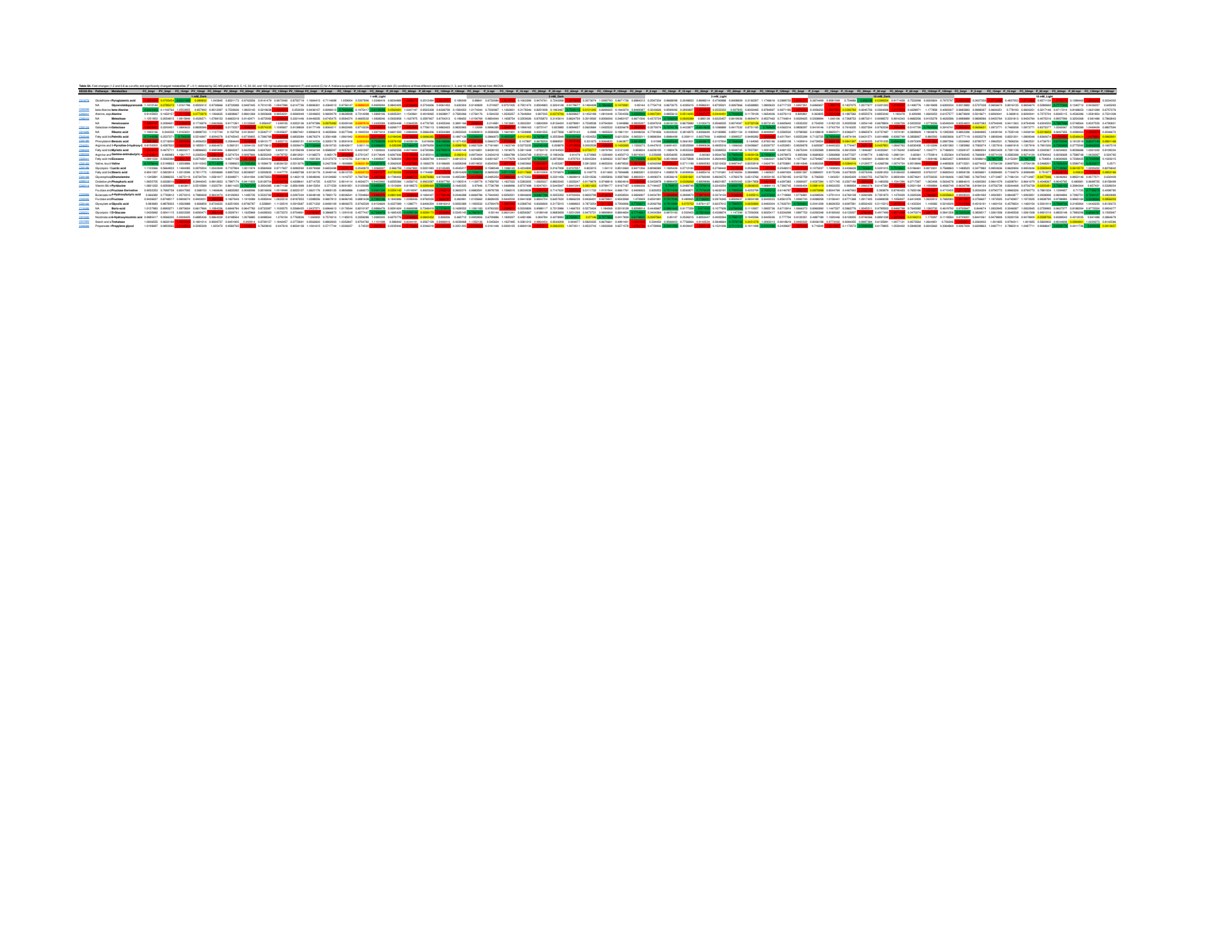

Metabolites	Treatment	Light	Concentrations	Time	Interactions
(6R)-5,6,7,8-Tetrahydrobiopterin	2.95E-10	0.21709614	8.47E-10	1.59E-36	0.998248641
5-Deoxy-5-methylthioadenosine	1.85E-08	0.0283078	4.53E-48	4.00E-25	0.842329105
Thymine	2.18E-06	0.12758237	0.057150633	1.64E-21	0.580639908
2-Deoxyadenosine	7.33E-08	0.8994166	2.72E-53	6.16E-20	0.913071542
Kinetin	2.44E-06	0.73082257	1.26E-12	3.47E-19	0.84409906
6-Furfurylaminopurine	1.36E-06	0.31597188	1.41E-12	5.44E-18	0.937485971
Inosine	7.80E-06	0.00029449	1.28E-47	5.64E-17	0.891109265
trans-4-Hydroxy-L-proline	0.183089059	0.0018439	6.38E-51	6.49E-16	0.494265868
Malonic acid	0.009143253	0.20842611	3.42E-16	7.27E-16	0.978218467
Isoleucine	3.03E-05	0.21415664	4.47E-58	2.12E-15	0.60958932
cis-4-Hydroxy-L-proline	0.003302905	0.06996235	1.56E-60	9.61E-15	0.445063737
Dihydrofolic acid	5.09E-05	0.08717428	2.71E-05	1.02E-14	0.478819098
Ferulic acid	1.19E-14	0.00756344	8.44E-19	3.80E-14	0.872985647
2-Deoxyguanosine-5-monophosphate	0.00018073	0.34812724	0.001969361	1.11E-13	0.993252588
Phenylalanine	5.47E-07	8.09E-19	1.38E-179	1.30E-12	0.23523849
Indole-3-butyric acid	0.00399157	0.69030735	2.10E-08	7.64E-12	0.555284985
Leucine	0.032068134	0.04655325	1.37E-65	1.06E-10	0.27334294
Zeatin	5.38E-06	0.23146029	4.97E-08	5.20E-10	0.738960706
Adenosine-3-monophosphate	0.000348176	0.07764249	1.35E-06	6.29E-10	0.976920419
Glutaric acid	0.021231293	0.00664952	1.05E-66	1.98E-09	0.267679721
L-Cystathionine	3.34E-07	0.51175836	0.83426022	2.35E-09	0.06987293
3-Methyl-L-histidine	0.658467596	0.87479972	3.37E-36	3.92E-09	0.046443591
O-Succinyl-L-homoserine	0.045574356	0.8329013	0.125160106	8.61E-09	0.146170288
Taurine	0.000262222	0.04341548	0.951141912	1.19E-08	0.13750876
Pyroglutamic acid	0.109292166	7.29E-20	4.23E-37	2.81E-08	0.951699198
Pyridoxal-5-phosphate	0.16046362	0.05254641	3.55E-08	4.21E-08	0.809870045
o-Hydroxycinnamic acid	0.002000387	0.97329519	4.21E-33	2.18E-07	0.096460586
Folic acid	0.143938231	0.10515898	0.000245025	2.98E-07	0.189959969
2-Deoxyadenosine-5-Monophosphate	4.38E-05	0.02577621	5.74E-25	3.14E-07	0.884618946
Methionine	0.062881346	0.29649541	7.92E-06	4.06E-07	0.537566435
N-Alpha-L Ornithine	2.00E-05	0.38332089	5.72E-05	7.26E-07	0.958765083
Acetylsalicylic acid	1.92E-05	0.83226143	3.83E-07	9.31E-07	0.005095981
Glutathione	2.82E-05	7.07E-05	1.04E-29	1.16E-06	0.892189107
Orotic Acid	0.169614	0.0794395	0.092659657	1.38E-06	0.769740454
Dehydroascorbic acid	0.469343791	0.05692256	2.59E-21	1.73E-06	0.508729856
Riboflavin	0.195958625	0.03973606	0.000942946	2.62E-06	0.796668115
4-HBA	0.024349598	0.37214839	0.000182469	5.25E-06	0.932107227
Fumaric acid	0.132835305	0.24605592	1.18E-05	7.98E-06	0.996684789
2-Hydroxyphenylacetic acid	0.393739001	4.86E-26	8.91E-20	8.71E-06	0.089452705
Catechin	0.658912427	0.00973348	5.02E-14	1.10E-05	0.049009536
Norepinephrine	0.000589651	0.25022394	0.204555308	1.62E-05	0.152502768
1-Methylnicotinamide	0.000287599	0.23879206	0.018901536	1.66E-05	0.268332398
Tyramine	0.000945935	0.41898465	3.74E-14	2.12E-05	0.886924103
3-Hydroxy 4-methoxycinnamic acid	1.79E-08	0.10382736	4.90E-13	2.14E-05	0.267292597
Epibrassinolide	0.013020816	0.58936489	0.099415495	3.00E-05	0.766468708
Hypotaurine	0.000190684	0.08697873	0.007704147	3.03E-05	0.000078504
Epicatechin	0.349177208	0.07723309	3.18E-17	3.35E-05	0.491539775
D-Glucosamine-6-phosphate	0.000100855	0.00187985	1.28E-08	3.38E-05	0.000303069
2-Aminoethylphosphonic acid	6.09E-06	0.13558566	0.805420195	4.17E-05	0.315319347
GSSG	0.003400712	0.00052936	7.44E-26	4.92E-05	0.810871093
Naringin	0.518842112	0.0039736	3.61E-09	5.20E-05	0.832324129
Homocysteine	0.161839656	0.03967824	1.62E-10	5.60E-05	0.477527326
4-Nitrophenol	0.041016827	1.44E-07	2.13E-08	6.30E-05	0.088847
Creatine	0.328133118	0.39959315	1.33E-21	6.57E-05	0.70995912
Callistephin chloride	0.07696242	0.03680039	2.17E-13	0.000100333	0.636762467
Cyanidin chloride	0.544703007	0.02325927	3.85E-10	0.000101821	0.070872511
Spermine	0.0355326	0.05685367	1.45E-44	0.00017655	0.785164745
3-Oxindolepropionic acid	0.139247586	0.19877936	0.02099829	0.000188552	0.324521936
Melibiose	0.020255542	0.26035808	1.60E-06	0.000196678	0.106239604
8-Nitro cGMP	0.002726920	0.57441573	2.00E-13	0.000214551	0.271257391
2-Hydroxycinnamic acid	0.57449011	1.33E-11	4.33E-07	0.000256976	0.134996211
Scopoletin	0.962738073	3.35E-127	2.08E-203	0.000262281	0.336578258
Methylmaloric acid	0.008872279	0.0004227	0.014222812	0.000298965	0.988117747
guanidine	0.010174706	0.04698463	0.001565134	0.000308521	0.963035295
Vanillin	0.046841853	0.91729556	1.12E-11	0.000328171	0.135358704
Triethanolamine	7.19E-06	2.82E-06	4.88E-10	0.000363479	0.975201006
Pelargonin chloride	0.218316341	0.00781363	2.94E-05	0.00041135	0.022355231
Luteolin-7-beta-glucoside	0.000245069	0.01904549	0.286561433	0.000445146	0.894207358
Eupatorin	0.12838447	3.44E-235	3.82E-201	0.000478205	5.84E-08
Galactinol dihydrate	0.264213492	0.00029689	1.16E-06	0.000685221	0.685932392
Methylthiobutyric acid	0.310814602	0.03294869	5.73E-53	0.0010836	0.883460951
L-Cysteine-S-sulfate	0.269394193	0.0178608	0.009053269	0.001697258	0.886295025
Uridine-5-monophosphate	0.074206964	0.00031426	4.70E-18	0.001734137	0.561990545
D-mannose-6-phosphate	0.019743088	0.00039822	2.45E-07	0.002593305	0.670576892
Shikimic acid	0.383614117	1.44E-06	0.00102211	0.00275311	0.015309932
Niacinamide	0.013878288	0.35182762	1.87E-06	0.003195033	0.177934355
Xanthophyll	0.347788848	0.32639361	5.82E-13	0.003396435	0.69418624
L-Malic Acid	0.59719364	4.75E-09	5.30E-17	0.003484724	0.589255727
Gamma-Glu-Cys	0.847410429	2.63E-05	0.000743734	0.003788729	0.025912782
2-Deoxyguanosine	0.001220198	0.01608801	2.04E-07	0.004113767	0.962627565
Alpha-D-Mannose-1-phosphate	0.916114699	0.08529618	0.008137958	0.004587305	0.982814512
Quercetin	0.613941845	1.79E-13	1.83E-66	0.004686234	0.320568741
Rosmarinic acid	0.389991273	0.04878741	6.85E-09	0.005157885	0.973032449
Isomaltose	0.38628565	0.18955575	4.49E-22	0.005334995	0.035022869
Cyclic adenosine diphosphate ribose	0.937640696	0.0441541	0.001232641	0.005878346	0.585007154
1-Methylhistamine	0.000192187	0.05925617	8.36E-21	0.006436632	0.061623113
Biochanin A 4-methylgeistein	0.021567867	1.76E-47	3.19E-51	0.00698643	0.867514763
2-Hydroxypyridine	0.586517224	0.40095458	0.002954106	0.008287439	0.371299167
2-Hydroxyisobutyric acid	0.121788496	0.02472993	0.002687168	0.008424269	0.100711052
Allantoin	0.035105562	0.19075961	2.60E-13	0.010382097	0.607267257
Zeatin riboside	0.687140094	0.84456416	2.14E-11	0.012247275	0.998604943
Citramalic acid	0.684445897	1.12E-25	1.80E-13	0.012691583	0.20946929
Anthranilic acid	0.139097891	0.48367257	3.44E-10	0.013252575	0.472694946
Asparagine	0.793746665	0.65188567	6.83E-39	0.014095524	0.47236991
Betaine aldehyde	0.072115293	0.00072531	0.995958074	0.016356814	0.357040235
3-Hydroxyanthranilic acid	0.294099699	0.00699288	2.12E-08	0.01729883	0.627487062
Riboflavin-5-Monophosphate	0.475176777	0.20317134	0.009195163	0.018581108	0.471900536
Caffeic acid	0.083206722	0.98240554	0.009923335	0.019087674	0.756826145
Rutin	0.000918465	0.00204309	3.95E-14	0.019147466	0.497054128
4-Hydroxy-3-methoxycinnamaldehyde	0.574431439	0.00554511	0.14871915	0.021193422	0.500050689
Salicin	0.070852977	0.92444533	1.06E-08	0.022341811	0.009875045
N-acetyl-D-mannosamine	0.204781268	0.66975537	0.000117279	0.025279552	0.957456916
2,3-Dihydroxybenzoic acid	0.771780916	4.68E-20	1.90E-22	0.02932767	0.323257569
1-Kestose	0.218474119	1.96E-06	7.48E-16	0.034161329	0.203738017
Agmatine sulfate	0.37951809	0.34063225	6.33E-87	0.03665242	0.167579487
O-Acetyl-L-serine	0.004653925	0.06555942	9.96E-98	0.039364854	0.009897215
L-Carnosine	0.004559637	0.19166138	2.42E-05	0.043402781	0.819941549
1-Methylnicotinamide	0.082200999	0.11771754	0.002211158	0.043456343	0.389394993
Thymidine	0.000109399	0.00279762	1.14E-19	0.043492114	0.720172583
3-Methylxanthine	0.293312964	1.08E-08	0.000464481	0.044024212	0.501222351
Dihydroorotic acid	0.147724228	0.58450784	6.45E-06	0.045600525	0.812937684
Alanine	0.192955243	0.53643368	4.25E-67	0.046906674	0.060018574
Ononin	0.863794364	0.18428719	1.61E-07	0.05447946	0.652699736
Hypoxanthine	0.332715267	0.01204901	5.08E-14	0.055455128	0.013604293
Serotonin	0.833999925	0.80099413	0.012530477	0.055677307	0.499255532
TriMethylamine-N-oxide	0.401507915	0.01026024	6.72E-35	0.05708113	0.004568001
Neohesperidin	0.250262272	5.36E-47	5.83E-13	0.062381349	0.064413649
Nicotinic acid	0.023916752	1.46E-08	0.055877971	0.063859537	0.042039099
Dihydrophaseic acid	0.015196787	0.00020244	1.16E-21	0.064780951	0.602182051
6-Gamma-dimethylallylaminopurine	0.322353622	2.75E-06	4.19E-05	0.067760566	0.262953597
Cadaverine	0.374656497	0.25131855	2.06E-61	0.068127616	0.099992806
Melatonin	0.026229994	0.00393177	0.000216867	0.069377848	0.287249108
Sphingosine-1-phosphate	8.59E-09	4.79E-05	2.62E-14	0.07105602	0.090337564
Apigenin	0.013368603	0.00098159	7.22E-07	0.072615461	0.305278734
Betaine	0.002086068	5.19E-08	2.08E-42	0.075157462	0.7871385
Maltose	0.04257829	2.85E-06	1.28E-06	0.076120442	0.152213377
Kaempferide	3.59E-09	0.74548034	7.00E-13	0.076957524	0.374279975
3, 4-Dihydroxy phenylaceticacid	0.176256953	9.87E-08	9.93E-16	0.080108289	0.518718666
Apigenin-7-glucoside	0.018701336	0.07706505	4.93E-05	0.081837098	0.925596619
Homogentisic acid	0.122395993	6.24E-13	1.51E-14	0.08214049	0.430898149
Adenosine	0.004304006	0.37194921	2.20E-68	0.084233071	0.228143092
Adenine	0.622403658	0.07396564	2.50E-09	0.084882785	0.496163189
Mandelic acid	0.862752362	7.73E-08	0.007210405	0.085491549	0.16629128
2-Guanidinopropionic acid	0.052423056	0.60197531	9.66E-06	0.09593675	0.38137293
Cyclic guanosine monophosphate	0.064985209	0.81606416	6.89E-12	0.10028194	0.682025399
Cytidine	0.803787998	0.34598517	8.09E-07	0.101053852	0.256021623
Cystathionine	0.380244875	0.8658135	1.54E-49	0.10790547	0.92449862
Genistein	0.112105333	0.47095341	8.88E-05	0.108500005	0.425214447
3-Indoleacetic acid	0.033897911	0.00569006	7.96E-06	0.119683753	0.304108207
Inosine-5-diphosphate	0.094732679	0.00168567	1.62E-67	0.12059614	0.021895618
Vanillic acid	0.900277593	1.05E-07	6.30E-13	0.121199767	0.906074631
Raffinose	0.171368762	0.00594056	1.79E-05	0.128467304	0.595550207
Cinnamic acid	0.513954496	0.00594073	3.36E-06	0.140322622	0.543976399
Hesperetin	0.000589453	7.06E-14	1.35E-18	0.145295536	0.696983362
Jasmonic acid	0.908586834	0.03348434	6.38E-37	0.1490776	0.402692264
Salicylic acid	0.288844491	0.02396645	1.88E-08	0.150771725	0.491769804
Uracil	0.813005033	0.98772788	0.311280876	0.152291644	0.756519738
Cellobiose	0.197418403	3.19E-06	3.73E-17	0.157528199	0.10864931
Glycolic acid	0.713985114	0.26527835	1.49E-11	0.163436211	0.009976334
Mesaconic acid	0.018445413	2.27E-12	1.94E-11	0.166925007	0.020689591
Alpha-lipoamide	0.089561039	0.00142095	4.62E-09	0.171975796	0.955035908
Threonine	0.859900271	0.69214254	2.53E-58	0.181848975	0.101958832
1,3,-Diaminopropane	0.2243820	0.11864341	1.29E-44	0.187309078	0.057902886
Vitexin	0.078844109	6.05E-09	1.28E-18	0.189503265	0.973808769
Acetylcholine	0.995615027	0.30441528	0.018347582	0.19274084	0.963903011
1,4-Diaminobutane_Putrescine	0.501827512	0.1709297	8.56E-27	0.21029671	0.481582191
1-Octacosanol	0.016514295	0.50427597	4.60E-19	0.213085124	0.196177173
Sarcosine	0.183132561	0.58920292	3.71E-68	0.215968649	0.058326564
Uridine	0.692916099	0.31143379	7.20E-09	0.218292526	0.941907344
Asparagine	0.938447112	0.3076995	1.03E-29	0.219673425	0.894976646
Tryptophan	0.227908919	1.52E-05	0.000114739	0.222847632	0.10338325
Diethanolamine	0.714508959	0.10781717	3.93E-54	0.223985553	0.208259118
Glutamine	0.621465847	0.77877928	9.71E-54	0.230230573	0.048963442
6-methoxycinnamic acid	0.57908469	7.73E-21	1.92E-17	0.233686536	0.309284002
4 Hydroxyphenylacetic acid	0.844666178	1.12E-08	2.30E-06	0.237012861	0.114642495
Abscisic acid glucose ester	0.996759809	0.12999573	1.36E-10	0.239648108	0.520248632
Homomethionine	0.732300802	0.04650003	0.000204216	0.246221051	0.024429546
Quercitrin	0.15095096	0.00017476	4.20E-13	0.262801261	0.616536712
O-phosphoserine	0.020458422	4.06E-05	0.033573861	0.263424005	0.960273753
Chlorophyll A	0.012614364	8.98E-06	7.18E-12	0.269872342	0.955364453
4-Imidazoleacrylic acid	0.255461818	0.13529956	1.28E-08	0.275337454	0.472284157
1-Methylhistidine	0.843451723	8.37E-05	6.93E-19	0.279229488	0.62857627
D-Ribose-5-phosphate	0.359862946	0.3518824	4.69E-09	0.284353487	0.193129124
Isoliquiritigenin	0.059282769	2.23E-06	1.38E-11	0.307750931	0.082274379
2-Deoxyinosine-5-triphosphate	0.49775534	0.06668367	0.002165895	0.309285814	0.785300888
D-Mannitol	0.290196053	2.43E-09	0.009383165	0.316167207	0.77794512
2,3-Pyridinedicarboxylic acid	0.086214514	0.14245194	1.59E-09	0.317009343	0.845867494
Guanosine	0.564954011	4.12E-16	7.37E-32	0.317277591	0.978576422
Guloric acid gammalactone	0.841165503	0.0486752	8.39E-11	0.325706142	0.209147061
2-Deoxyribose-5-phosphate	0.015901433	0.00142822	7.44E-06	0.332287623	0.582348537
Gibberellin A3	0.966001329	3.77E-08	6.26E-05	0.335419111	0.275406095
7-Hydroxyflavone	0.305697622	0.0001138	0.000215544	0.339739167	0.947057099
Carnitine	0.056272996	0.02089846	2.42E-15	0.342659394	0.001289656
Eriodictyol	0.418988271	0.03972031	8.69E-15	0.358514466	0.243015351
Curcumin	0.59686135	0.71193385	0.000197821	0.359099463	0.572816514
3,4-Hydroxyphenylpropionic acid	0.001074926	0.01383304	4.12E-05	0.364713718	0.663256625
Lignoceryl alcohol	0.039148135	2.72E-09	1.21E-14	0.366044787	0.120252789
L-Homocysteine	0.451590125	0.96413407	3.49E-15	0.36986067	0.737746655
Caffeine	0.004283676	0.01727606	0.001213299	0.373908101	0.36455068
Guanine	0.529487797	0.29072733	5.44E-06	0.379378259	0.66057478
Capsaicin	2.93E-22	3.74E-15	1.23E-14	0.385272669	0.560636191
Alpha-lactose	0.045649146	0.76311016	0.000100511	0.385533519	0.133385241
Glucuronic acid	0.369486326	0.14449449	1.13E-07	0.385598533	0.113127405
Cysteine	0.103643647	5.79E-18	1.94E-29	0.393796147	0.663831749
2-Deoxyinosine	0.127620632	0.00113551	0.000244612	0.400862805	0.159447784
Luteolin	0.035606801	0.00019371	0.002313326	0.419969484	0.308454842
Naringenin	0.078276703	0.01933987	2.95E-32	0.42364713	0.265907039
D-Glucose-6-phosphate	0.957131149	0.2236232	0.00932779	0.43093684	0.854320625
Syringic acid	0.333001495	3.66E-14	6.12E-20	0.438007287	0.130341315
L-Citrulline	0.010127276	0.37720987	4.26E-53	0.445582756	0.149024158
Aspartic Acid	0.863000791	0.00058502	4.18E-15	0.449347749	0.090816531
Sphingosine	0.016806717	0.12256333	1.74E-20	0.46992057	0.916634246
Methyl indoleacetate	0.592982211	0.72483329	6.02E-06	0.470967115	0.887847316
Spermidine	0.130364233	0.02097556	3.49E-66	0.477708706	0.542887399
Arbutin	0.105924633	9.19E-07	1.77E-14	0.480788097	0.269327763
Tryptamine	0.131203329	0.69496618	0.466836962	0.483420585	0.103096228
4-Methyl-5-Thiazolethanol	0.024309206	0.95417575	1.37E-08	0.485093676	0.269361138
Histidine	0.926747456	0.13529185	2.50E-06	0.505137909	0.159794603
Tyrosine	0.797594621	0.52779491	1.22E-42	0.509720428	0.455932218
cis-Aconitic acid	0.591806267	0.53303449	8.53E-12	0.511259831	0.007057477
Pyridoxine	0.632319042	0.92162204	1.28E-07	0.525867545	0.378353333
Indole carboxylic acid	0.98130694	0.71578556	2.78E-06	0.53179605	0.579822083
Glutamic acid	0.059827935	0.77053465	2.21E-59	0.552782362	0.037566432
Cysteamine	0.00788726	0.68349506	7.20E-07	0.554695176	0.960013966
Treponone	0.393561024	1.63E-17	0.090690508	0.556090619	0.73981393
5,6-Dihydrouracil	0.352459943	0.55655068	9.66E-26	0.563149092	0.592125556
Lysine	0.565147504	0.71726692	1.26E-55	0.565488007	0.046877779
Dodecanoic acid	0.000746389	0.6197203	0.007351899	0.56975292	0.276553314
Pyrocatechol	0.001692182	0.00013019	2.71E-70	0.607783945	0.526244363
Sodium-3-methyl-2-oxobutyrate	0.104285693	2.52E-58	1.74E-77	0.615646927	0.551735447
L-Methionine sulfone	0.384546073	0.02095153	0.000563714	0.620645664	0.64655632
2-Isopropylmalic acid	0.7274905	0.04597331	0.000597798	0.63396749	0.767131811
Coumaric acid	0.000311551	3.26E-20	0.000043717	0.637642415	0.050472937
3-Methyladenine	0.071243866	0.68271018	1.45E-10	0.660295942	0.451403522
Arginine	0.13455201	0.40705539	2.79E-35	0.669659016	0.011557601
Pimelic acid	0.748547226	0.4564169	0.125619442	0.669935828	0.926935545
Histamine	0.016218522	0.00094267	0.000163124	0.707614905	0.084430322
L-Ornithine	0.26787003	0.03095593	2.00E-33	0.739408121	0.33847488
Succinic acid	0.409792506	2.93E-23	7.87E-21	0.748690005	0.121562593
I-ACC	0.097180355	0.62163878	9.86E-42	0.76161201	0.52710455
Nicotinamide	0.375539648	0.11366974	1.02E-10	0.762467023	0.252653379
Thiamine	0.360715266	0.00270015	3.23E-09	0.766024711	0.060924254
Ethylmalonic acid	0.492983126	0.18409024	1.60E-54	0.771498738	0.41419893
N,N-Dimethylglycine	0.080865333	0.688102	1.18E-06	0.77487914	0.053750134
Cytosine	0.324664455	0.36149503	0.011580032	0.82809903	0.596022853
3, 4 Dihydroxyphenylalanine	0.001295656	0.01791824	1.80E-08	0.833093395	0.100474485
5-Methylcytosine	0.048233658	0.22745276	0.000995006	0.835629545	0.007059417
GSH	0.715741454	1.20E-05	9.01E-10	0.878277892	0.321810693
Liquiritigenin	0.745076656	0.4906204	5.19E-09	0.91129978	0.186561432
Chlorogenic acid	0.943532793	0.03133008	1.01E-11	0.911346349	0.573948216
N-formyl-L-methionine	0.185386591	0.13765237	2.19E-05	0.915184266	0.661596093
6-Hydroxyflavone	0.187953036	0.00015667	4.23E-16	0.925125895	0.191175293
Xanthine	0.821927508	1.30E-07	3.59E-06	0.936057512	0.738055115
6,7 Dihydroxycoumarin	0.100455929	5.23E-05	2.82E-05	0.964444026	0.561553298

Table S6. Pathways enrichment of STEM analysis patterns of metabolite changes across light conditions (light and dark), concentrations (1, 3, and 10 mM). Numbers indicate the number of metabo

Pathway Name	Total	Comp Hits	P (<0.1)	Pathway Name	Total	Comp Hits	P (<0.1)
**Dark**				**Light**			
**1mM, 75 Metabolites, Model-6**				**1mM, 80 Metabolites, Model-6**			
Purine metabolism	61	8	0.011333	Flavone and flavonol biosynthesis	9	3	0.011465
Nicotinate and nicotinamide metabolism	12	3	0.021118	Flavonoid biosynthesis	43	6	0.030802
Flavone and flavonol biosynthesis	9	2	0.075761	Tyrosine metabolism	18	3	0.077222
Riboflavin metabolism	10	2	0.091598				
**1mM, 50 Metabolites, Model-7**				**1mM, 45 Metabolites, Model-9**			
Arginine and proline metabolism	38	5	0.0096586	Arginine and proline metabolism	38	4	0.029801
Alanine, aspartate and glutamate metabolism	22	3	0.040807	beta-Alanine metabolism	12	2	0.053335
beta-Alanine metabolism	12	2	0.0656	Pantothenate and CoA biosynthesis	14	2	0.070658
Aminoacyl-tRNA biosynthesis	67	5	0.085858				
**1mM, 49 Metabolites, Model-8**				**1mM, 26 Metabolites, Model-0**			
Flavone and flavonol biosynthesis	9	3	0.0037016	Arginine and proline metabolism	38	6	4.01E-05
Taurine and hypotaurine metabolism	5	2	0.013179	Aminoacyl-tRNA biosynthesis	67	7	1.29E-04
Pyrimidine metabolism	38	4	0.052679	Alanine, aspartate and glutamate metabolism	22	3	0.0069438
Valine, leucine and isoleucine biosynthesis	26	3	0.072774	Glutathione metabolism	26	3	0.011162
				beta-Alanine metabolism	12	2	0.019605
				Nitrogen metabolism	15	2	0.030145
				Valine, leucine and isoleucine biosynthesis	26	2	0.08242
**3mM, 92 Metabolites, Model-6**				**3mM, 65 Metabolites, Model-6**			
Alanine, aspartate and glutamate metabolism	22	6	0.0023317	Flavone and flavonol biosynthesis	9	3	0.006003
Arginine and proline metabolism	38	7	0.010677	Indole alkaloid biosynthesis	7	2	0.03618
Tyrosine metabolism	18	4	0.027555	Nicotinate and nicotinamide metabolism	12	2	0.09847
Aminoacyl-tRNA biosynthesis	67	9	0.029767				
Valine, leucine and isoleucine biosynthesis	26	4	0.089761				
Glutathione metabolism	26	4	0.089761				
One carbon pool by folate	8	2	0.094866				
**3mM, 73 Metabolites, Model-9**				**3mM, 48 Metabolites, Model-9**			
Flavone and flavonol biosynthesis	9	5	3.27E-05	Glutathione metabolism	26	5	0.0012788
Flavonoid biosynthesis	43	5	0.064427	Vitamin B6 metabolism	11	2	0.049471
Purine metabolism	61	6	0.086684	Starch and sucrose metabolism	30	3	0.075203
**3mM, 31 Metabolites, Model-7**				**3mM, 40 Metabolites, Model-7**			
beta-Alanine metabolism	12	3	0.0021797	Histidine metabolism	16	3	0.010181
Pyrimidine metabolism	38	4	0.010179	Arginine and proline metabolism	38	4	0.023143
Fructose and mannose metabolism	16	2	0.051412	Cysteine and methionine metabolism	34	3	0.075568
Amino sugar and nucleotide sugar metabolism	41	3	0.067009				
**10mM, 104 Metabolites, Model-6**				**10mM, 64 Metabolites, Model-9**			
beta-Alanine metabolism	12	5	0.0011331	Taurine and hypotaurine metabolism	5	2	0.017068
Arginine and proline metabolism	38	9	0.0013976	Isoquinoline alkaloid biosynthesis	6	2	0.024892
Isoquinoline alkaloid biosynthesis	6	3	0.0070112	Tyrosine metabolism	18	3	0.039951
Alanine, aspartate and glutamate metabolism	22	5	0.020544	Valine, leucine and isoleucine biosynthesis	26	3	0.0998
Cysteine and methionine metabolism	34	6	0.037351				
Glutathione metabolism	26	5	0.040376				
Tyrosine metabolism	18	4	0.040751				
Aminoacyl-tRNA biosynthesis	67	9	0.058315				
Glycine, serine and threonine metabolism	30	5	0.068948				
**10mM, 29 Metabolites, Model-8**				**10mM, 63 Metabolites, Model-6**			
Valine, leucine and isoleucine degradation	34	4	0.0052948	Flavone and flavonol biosynthesis	9	3	0.0057129
Pantothenate and CoA biosynthesis	14	2	0.035302	Flavonoid biosynthesis	43	6	0.0098105
Fructose and mannose metabolism	16	2	0.045324	beta-Alanine metabolism	12	2	0.095567
**10mM, 28 Metabolites, Model-9**				**10mM, 30 Metabolites, Model-7**			
Pentose phosphate pathway	18	2	0.042459	Purine metabolism	61	4	0.035261
Flavonoid biosynthesis	43	3	0.043036				
Galactose metabolism	26	2	0.08242				
Taurine and hypotaurine metabolism	5	1	0.089894				
